# Neuropilin-1 Expression Associates with Poor Prognosis in HNSCC and Elicits EGFR Activation upon CDDP-Induced Cytotoxic Stress

**DOI:** 10.3390/cancers13153822

**Published:** 2021-07-29

**Authors:** Virginia Napolitano, Daniela Russo, Francesco Morra, Francesco Merolla, Silvia Varricchio, Gennaro Ilardi, Rosa Maria Di Crescenzo, Francesco Martino, Massimo Mascolo, Angela Celetti, Luca Tamagnone, Stefania Staibano

**Affiliations:** 1 Dipartimento di Scienze della Vita e Sanità Pubblica, Università Cattolica del Sacro Cuore, 00168 Roma, Italy; virginia.napolitano@unicatt.it (V.N.); luca.tamagnone@unicatt.it (L.T.); 2 Dipartimento di Scienze Biomediche Avanzate, Unità di Anatomia Patologica, Università degli Studi di Napoli “Federico II”, 80131 Napoli, Italy; daniela.russo@unina.it (D.R.); silvia.varricchio@unina.it (S.V.); gennaro.ilardi@unina.it (G.I.); rosamaria.dicrescenzo@unina.it (R.M.D.C.); francesco.martino@unina.it (F.M.); massimo.mascolo@unina.it (M.M.); staibano@unina.it (S.S.); 3 Istituto di Endocrinologia e Oncologia Sperimentale “Gaetano Salvatore”, CNR, 80131 Napoli, Italy; francesco.morra@unina.it (F.M.); celetti@unina.it (A.C.); 4 Dipartimento di Medicina e Scienze della Salute “V. Tiberio”, Università degli Studi del Molise, 86100 Campobasso, Italy; 5 Fondazione Policlinico “A. Gemelli”, IRCCS, 00168 Roma, Italy

**Keywords:** NRP-1, EGFR, HNSCC, cisplatin

## Abstract

**Simple Summary:**

NRP-1, a co-receptor of the EGFR, represented an interesting candidate to investigate in HNSCC, as Cetuximab, in combination with radio and chemotherapy, provided the first targeted therapy scheme approved by the FDA as a standard of care for patients with recurrent or metastatic HNSCC. High levels of NRP-1 expression significantly correlated with a shorter overall survival in both Oral Squamous Cell Carcinoma and Oropharyngeal Squamous Cell Carcinoma diagnosed patients, suggesting a prognostic role for this protein. In HNSCC cell lines in vitro experiments, NRP-1 sustained EGFR activation upon CDDP exposure, together with activation of downstream MAPK/AKT pathways. Furthermore, NRP-1 modulated the responsiveness to CDDP treatment.

**Abstract:**

Head and neck squamous cell carcinoma (HNSCC) includes a group of aggressive malignancies characterized by the overexpression of the epidermal growth factor receptor (EGFR) in 90% of cases. Neuropilin-1 (NRP-1) acts as an EGFR co-receptor, enhancing, upon ligand stimulation, EGFR signaling in several cellular models. However, NRP-1 remains poorly characterized in HNSCC. By utilizing in vitro cellular models of HNSCC, we report that NRP-1 is involved in the regulation of EGFR signaling. In fact, NRP-1 can lead to cisplatin-induced EGFR phosphorylation, an escape mechanism activated by cancer cells upon cytotoxic stress. Furthermore, we evaluated Neuropilin-1 staining in tissue samples of an HNSCC case series (*n* = 218), unraveling a prognostic value for the Neuropilin-1 tissue expression. These data suggest a potential role for NRP-1 in HNSCC cancer progression, expanding the repertoire of signaling in which NRP-1 is involved and eliciting the need for further investigations on NRP-1 as a suitable target for HNSCC novel therapeutic approaches.

## 1. Introduction

Head and neck squamous cell carcinoma (HNSCC) represents the sixth most common cancer worldwide, accounting for more than 550,000 new cases and 380,000 deaths annually [1]. HNSCC encompasses a group of malignancies arising in the oral cavity, oropharynx, larynx, and hypopharynx. Until now, the risk stratification for HNSCC has been mainly based on the stage and the high-risk human papillomaviruses (HPVs) infection status in the case of oropharyngeal cancers. In particular, the chronic infection by high-risk HPVs is a recognized cause of a large part of oropharyngeal cancers and, importantly, correlates with a more favorable outcome [2,3]. Up to now, the treatment choice for HNSCC patients is largely determined by the tumor stage at diagnosis. Commonly, HNSCC at an early stage (I or II) is treated with local therapy, such as surgical removal and/or radiation therapy, while advanced disease (stage III or IV) requires multimodality treatment with surgery, radio, and/or chemotherapy [4]. Cisplatin is one of the chemotherapeutic drugs most commonly used today for advanced HNSCC; a combination with Cetuximab (monoclonal antibody targeting EGFR) has been approved as first-line treatment for recurrent/metastatic HNSCC [5], representing, to date, the only targeted therapy approved for the treatment of HNSCC [5,6]. The EGFR protein is reported to be overexpressed in most HNSCC [7]. Alterations of EGFR represent one of the major events in HNSCC, EGFR-activating mutations are not frequently detected, but EGFR gene amplification is reported in 24–58% of HNSCC, supporting its role as a driver gene in HNSCC [8,9,10]. Neuropilin-1 (NRP-1), as EGFR co-receptor, resulted in an interesting candidate to explore in HNSCC. NRP-1 is a transmembrane glycoprotein with a molecular mass of 130 kDa, composed of a large extracellular region, including the ‘a1/a2’ (CUB), ‘b1/b2’ (FV/FVIII), and ‘c’ (MAM) domains; a transmembrane domain; and a short cytoplasmic region [11]. Neuropilin-1 was originally discovered as a regulator of the nervous system development, acting as semaphorin (SEMAs) co-receptors in a complex with plexins [12] and later as a receptor for some members of Vascular Endothelial Growth Factor (VEGFs) family [13]. Interestingly, in recent years, the role of NRP-1 in enhancing tyrosine kinase receptors signaling upon ligands activation, such as Hepatocyte Growth Factor(HGF), Platelet-derived Growth Factor (PDGF), and Epidermal Growth Factor (EGF), has been investigated. Then, NRP-1 results implicated in multiple oncogenic paths, such as cellular proliferation, survival, invasion, and migration. Recently, it has been reported that NRP-1 can exert ligand-dependent control of EGFR signaling [14,15]. Upon EGF or TGF-α stimulation, NRP-1 interacts with the EGF receptor, controlling its clustering on the cell surface and also the endocytosis process, then eliciting the activation of the downstream effectors, AKT and MAPK [16]. In this work, in a study population of HNSCC clinical specimens, we explored the tissue expression by immunohistochemistry of NRP-1 and performed a statistical analysis with patients’ prognoses. Additionally, in in vitro experiments, we found that NRP-1 could affect HN cells responsiveness to CDDP treatment, supporting the EGFR activation upon CDDP treatment. Intriguingly, we propose, for the first time, NRP-1 as a molecule capable of sustaining the cisplatin-induced EGFR activation in a ligand-independent manner, a known cellular mechanism reported in response to chemotherapeutic stress.

## 2. Materials and Methods

### 2.1. Cell Culture

The HN, CAL27, CAL33, HN6, and HN13 cell lines were grown in Dulbecco’s Modified Eagle Medium (DMEM), supplemented with 10% fetal bovine serum (Sigma-Aldrich, Gillingham, UK), 1% of 200 mM l-glutamine (Autogen Bioclear, Wiltshire, UK), 1% of 10.000 units Penicillin, and 10 mg/mL Streptomycin (Sigma-Aldrich, Gillingham, UK) and incubated at 37 °C in 5% CO${}_{2}$, as previously described [17].

### 2.2. Drugs

Cis-Diammineplatinum(II) dichloride (cisplatin) (C2210000) was provided by Sigma-Aldrich S.r.l. (Sigma-Aldrich, Gillingham, UK). The caspase-3 inhibitor Z-VAD-FMK was from Merk Millipore (Merck Millipore Corporation, Burlington, MA, USA).

### 2.3. Cell Viability

For in vitro viability assays, cells were seeded (1000 cells/well, three technical replicates) and, after 24 h, treated with different cisplatin doses (2.5; 5; 10; 20 μM) or vehicle (Phosphate-buffered saline, PBS) for 72 h. After the end of treatment, the cell medium was discarded, the CellTIter-Glo reagent (Promega Inc., Madison, WI, USA) was added, and the plate was incubated for 10 min at room temperature. The luminescence was measured in a Multilabel Reader (PerkinElmer, Waltham, MT, USA).

### 2.4. Gene Silencing

To achieve stable knockdown, NRP-1 expression was silenced in tumor cells by transducing them with shRNA-expressing lentiviral constructs. An NRP-1-targeting sequence (GAGAGGUCCUGAAUGUUCC) was inserted in the lentiviral transfer plasmid pCCLsin.PPT.hPGK.GFP.Wpre in the frame of a sequence driving the transcription of a short-hairpin RNA under control of the H1 promoter. Control shRNA (pLKO) was generated by introducing 4 base substitutions in the NRP-1-targeting sequence (GATAGGTCATGACTGCCC). We silenced NRP-1 expression by means of a puromycin selectable lentiviral construct TRCN0000323055, provided by Sigma-Aldrich.

### 2.5. Western Blot Analysis

Whole protein extracts were prepared using LB buffer and quantified using the BCA Protein Assay kit (Pierce, Rockford, IL, USA). Primary antibodies, anti-NRP-1 (ab81321) and anti-pEGFR (Tyr1068) (ab5644), were from Abcam (Cambridge, UK); anti-Vinculin (1931) and anti-Tubulin (T6199) were from Sigma; anti-MAPK (4695s), anti-pMAPK (4370s), anti-pAKT (9271s), anti-AKT (9272s), and anti-EGFR (1005:sc-03) were from Cell Signaling. Secondary antibodies were from Amersham, UK. The detection was performed with the ECL system (Amersham, UK).

### 2.6. RNA Isolation and Real-Time PCR

Total RNA from tumor cell lines or tissues was isolated with the RNeasy Mini Kit (Qiagen, Germantown, MD, USA), according to the manufacturer’s instructions [18]. cDNA preparation was conducted according to standard procedures, using M-MLV Reverse Transcriptase (Promega, Madison, WI, USA) and oligo-dT primers (Promega, Madison, WI, USA). Gene expression was measured using the following Taqman gene-specific probes from Thermo Fisher Scientific: NRP-1 (Hs00826128_m1), EGFR (Hs00193306m1), and the housekeepers GAPDH (Hs04420632_g1), and β-actin (Hs99999903_m1) [19].

### 2.7. TMA and IHC

**Figure 1:**
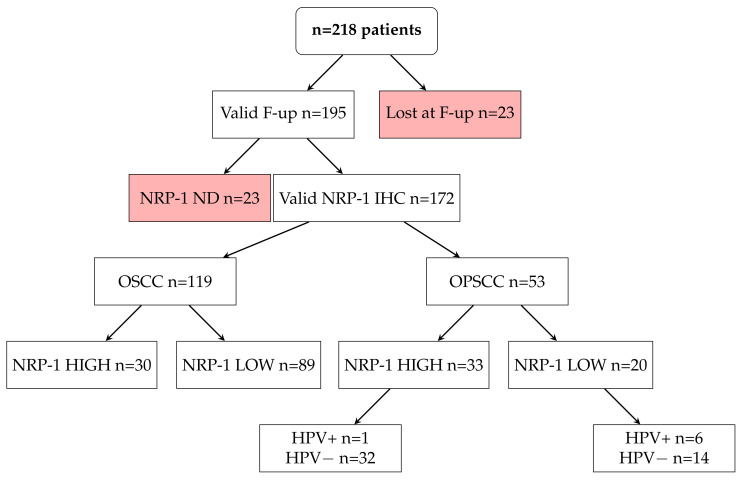
Study population composition and distribution of samples according to the tumor site and NRP-1 positivity groups. OPSCC samples were further classified according to HPV positivity. Nrp: Neuropilin-1; ND: not detectable; OSCC: Oral Squamous Cell Carcinoma; OPSCC: Oropharyngela Squamopus Cell Carcinoma.

Formalin-Fixed and Paraffin-Embedded Tumor samples were obtained from the archives of the Pathology Unit of the University of Naples “Federico II”. To the aim of our study, We selected 218 HNSCC tumor samples (149 OSCC and 69 OPSCC, of which 8 HPV-positive) [20]. A total of 23 patients were lost at follow-up, and 23 samples were classified as not valid at the IHC-staining evaluation. The final study population was of 172 samples, with complete follow-up and valid NRP-1 IHC staining (119 OSCC and 53 OPSCC). The HPV positivity was confirmed through p16 immunostaining and HPV genotyping by INNO LiPA. In total, 7/53 OPSCC tumor samples were HPV positive, and 46/53 were HPV negative (Figure 1). The Sections (4 μM) were stained with hematoxylin and eosin (H&E). A Tissue Micro-Array (TMA) was built using the most representative areas from each selected paraffin block. Using a semi-automated tissue arrayer (Galileo TMA, Milan, Italy), 1 mm tissue cores were punched from morphologically representative tissue areas of each donor block and placed into one recipient paraffin block (3 × 2.5 cm) [21]. The immunohistochemical stainings were performed with anti-NRP-1 ab (Abcam-Cambridge, Cambridge, UK) as described [22].

### 2.8. Digital Image Analysis and Statistical Analysis

H&E-stained and IHC-stained glass slides were digitalized at 40× using the Leica Aperio AT2 slide scanner (Leica Biosystems, Wetzlar, Germany) [23]. WSI images in .svs file format were analyzed with the QuPath platform. Classification was performed applying a Random Tree classifier [24]. The staining vector signal intensity was assessed and quantified to obtain a H-Score for NRP-1 tissue expression in both OSCC and OPSCC samples. NRP-1 H-Score values were categorized for each tumor site (OSCC and OPSCC) into low and high-expression groups; the threshold for categorization was selected via ROC curve analysis for the OS (Overall Survival) outcome (Figure 1). For TCGA dataset analysis, data were retrieved from the TCGA website, and mRNA levels of the NRP1 gene were analyzed and thresholded using kmplot.com analysis tools [25] (Last access on 14 July 2021).

### 2.9. Caspase Assay

Cells were plated at 1000 cells/well, three technical replicates, and treated with cisplatin at 20 μM for 24 h. Apoptosis was quantified by measuring Caspase 3/7 activation using the Caspase-Glo 3/7 assay (Promega, Madison, WI, USA), according to the manufacturer’s instructions.

### 2.10. Colony Forming Assay

Cells were plated at the density of 1 × 10^4^ cells/well into six-well plates, incubated for 24 h, and then treated with different doses of cisplatinum. After incubation for 10 days, colonies formed were stained with crystal violet, then scored and plotted following normalization versus control (a population of more than 30 cells was scored as one survivable colony, and plots show mean colony counts ± standard errors). The colonies’ counting was performed at the optic microscope and by using the open source software ImageJ-NIH.

## 3. Results

### 3.1. NRP-1, NRP-2 and EGFR Expression Levels in HNSCC Cell Lines

**Figure 2:**
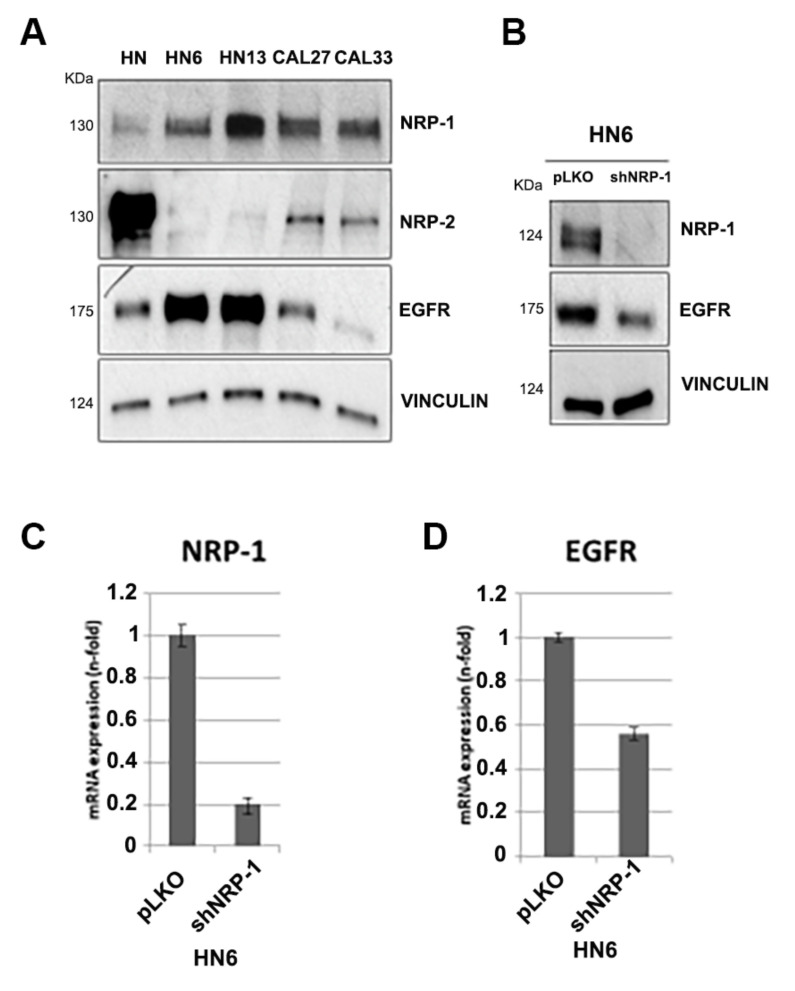
(**A**) NRP-1, NRP-2, and EGFR expression levels in HNSCC cell lines: Western blot (WB) analysis of NRP-1, NRP-2, and EGFR expression detected by specific antibodies, as indicated. (**B**) In HN6 cells, the silencing of NRP-1 affects EGFR activation as detected in the WB. Vinculin in A and B has been utilized as loading control. (**C**,**D**) NRP-1 and EGFR relative expression was assessed by RealTime-PCR in HN6 cells transfected with pLKO, as control, or with the shNRP-1 vector.

To characterize the functional role of NRP-1 in HNSCC and to investigate the drug sensitivity, we utilized in vitro HNSCC cell lines. First, in HN, HN6, HN13, CAL27, and CAL33 cells, we assessed the expression levels of NRP-1, NRP-2, and EGFR (Figure 2A) by Western blot analysis. NRP-1 expressed at variable levels in all of the analyzed cells. On the contrary, NRP-2, highly expressed in HN cells, was observed at low levels in CAL27 and CAL33 and was almost undetectable in HN6 and HN13 cells. Thus, in our cell systems, the level of NRP-2 seemed to inversely correlate with the expression level of NRP-1. The EGFR protein was found at high levels in HN6 and HN13 cells, as already reported [26]. Then, we wanted to investigate whether NRP-1 knockdown might impact the cisplatin sensitivity of our cells. To this aim, we performed NRP-1 depletion, revealing that NRP-1 silencing in HN6 cells determined a decrease in the expression levels of EGFR (Figure 2B), which was presumably dependent on a negative regulation at the trascriptional level, as the RealTime-PCR data sustain (Figure 2C,D). Similar cisplatin-induced EGFR activation was obtained in CAL33 and HN13 cells (see Section 3.2).

### 3.2. NRP-1 Sustains EGFR Activation upon CDDP Exposure

**Figure 3:**
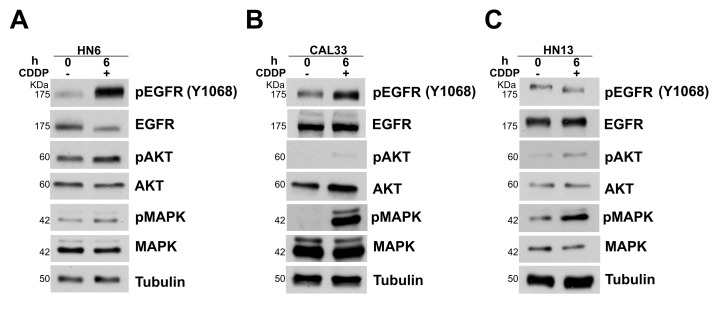
The activation of the EGFR pathway following CDDP exposure in HN6 (**A**), CAL33 (**B**), and HN13 (**C**) cells.

**Figure 4:**
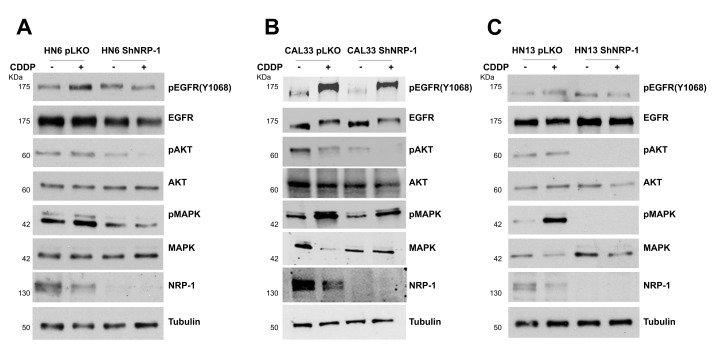
NRP-1 sustains CDDP-induced EGFR activation. Western blot detection of pEGFR (Y1068), and EGFR in HN6 (**A**), CAL33 (**B**), and HN13 (**C**) cells silenced for Neuropilin-1 (shNRP-1) or control (pLKO) following treatment with cisplatinum for 6 h.

CDDP is one of the most common drugs used for the treatment of advanced head and neck squamous cell carcinomas. In order to evaluate whether the CDDP-induced activation of EGFR may be controlled by NRP-1, we first treated HN6, HN13, and CAL33 cells with CDDP, observing the highest increase in the levels of EGFR phosphorylation after 6 h of CDDP treatment (Figure 3A–C). A weaker EGFR phosphorylation was detected in HN13 cells, which could be ascribed to the reported substitution of the histidine residue-773 with tyrosine (H773Y) in the kinase receptor expressed in these cells (Table 1). Interestingly, following NRP-1 depletion, we observed a significant impairment of CDDP-induced EGFR activation in HN6 and CAL33 cells, together with the reduced activation of downstream MAPK/AKT pathways (Figure 4A,B). In HN13 cells, basally showing a poorer response to CDDP, NRP-1 silencing slightly affected the CDDP-induced EGFR phosphorylation; however, this was sufficient to impact downstream effectors activation (Figure 4C). EGFR activation turned on its downstream signaling pathways, as indicated by the MAPK and AKT phosphorylation increase. Notably, the latter was abrogated upon treatment with a selective EGFR inhibitor, underscoring the importance of this tyrosine kinase in the adaptive response of cancer cells to CDDP (Supplementary Figure S1).

### 3.3. NRP-1 Affects Responsiveness to CDDP Treatment

**Figure 5:**
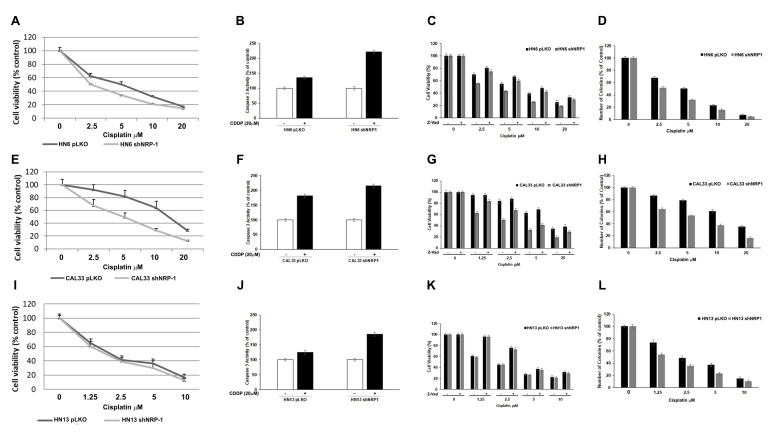
Cell viability of the NRP-1-silenced HNSCC cells compared to the control cells upon cisplatin treatment. Three independent experiments were performed to calculate the mean and standard deviation. (**A**,**E**,**I**: HN6, CAL33, and HN13, respectively). Caspase 3 activity was assayed in HN6, CAL33, and HN13 cells (**B**,**F**,**J**, respectively) transfected with the control vector (pLKO) or with ShNRP1 in the presence or not of cisplatin (20 μM) for 24 h. The plotted values represent the mean ± s.e.m. of three independent experiments. At 72 h, the CDDP (20 μM) effect on cell viability was modulated by NRP1 silencing and rescued by the addition of Z-VAD-FMK to the culture medium (**C**,**G**,**K**: HN6, CAL33, and HN13, respectively). Histograms represent the percentage of colonies consisting of at least 30 cells and normalized on untreated cells, which were counted following clonogenic assays performed in pLKO or ShNRP1 HN6, CAL33, and HN13 cell lines that were treated with different doses of CDDP for 10 days (**D**,**H**,**L**). Error bars indicate the standard error mean derived from three independent experiments.

In order to investigate whether NRP-1 levels could impact the CDDP sensitivity HN6, HN13, and CAL33 cells, wild-type or silenced for NRP-1, were treated with a range of doses of CDDP (0; 2.5; 5; 10; 20 μM) for 72 h. The viability of the cells was assessed by a cell titer assay. Compared to controls, NRP-1 silencing determined an enhanced sensitivity to CDDP in HN6 cells (shNRP-1 IC50 = 2.5 μM vs. pLKO IC50 = 5.5 μM) and in CAL33 cells (shNRP-1 IC50 = 5 μM vs. pLKO IC50 = 12 μM) (Figure 5A,E). However, in HN13 cells, a significant difference in the IC50 between the shNRP-1 vs. pLKO cells was not appreciated, suggesting that NRP-1 might have a major impact on CDDP sensitivity in the EGFR wild-type (CAL33) and EGFR amplified (HN6) HNSCC cells (Figure 5I) (Table 2). Furthermore, CDDP treatment increased the cell death in NRP-1 silenced cells by inducing apoptotic cell death, as shown by different assays. NRP-1 depletion determined caspase 3 activation upon CDDP treatment (20 μM) (Figure 5B,F,J). Moreover, the pan-caspase inhibitor Z-VAD-FMK impaired the CDDP-induced cytotoxicity in the HN6, HN13, and CAL33 cells (Figure 5C,G,K). Finally, in these cells, the downregulation of NRP-1 affected the colony formation ability following 10 days of exposure to different doses of CDDP, as indicated (Figure 5D,H,L).

### 3.4. NRP-1 Expression Level Is a Prognostic Marker for HNSCC Patients

**Figure 6:**
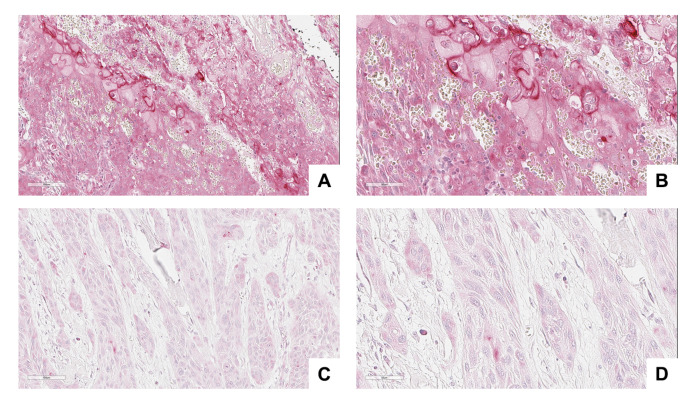
IHC evaluation of Neuropilin-1 tissue expression. (**A**,**B**): A representative case of Neuropilin-1 high expression. (**C**,**D**): A representative case of Neuropilin-1 low expression. (Virtual slide magnifications: (**A**,**C** 20×; **B**,**D** 40×; scale bars are shown).

**Figure 7:**
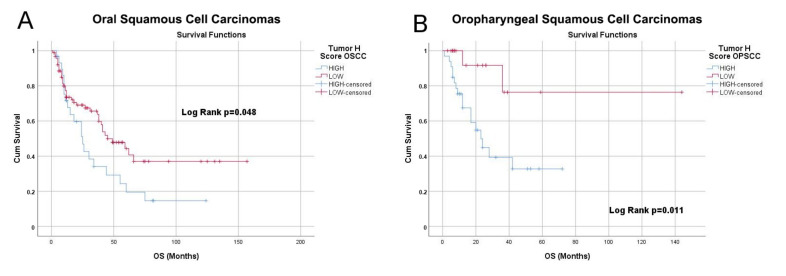
Kaplan–Meier Survival Curves analysis. (**A**) HIGH and LOW NRP-1 expression survival curves were compared in OSCC samples (*p* = 0.048 at Log Rank test for significance). (**B**) HIGH and LOW NRP-1 expression survival curves were compared in OPSCC samples (*p* = 0.011 at Log Rank test for significance).

In order to evaluate the role of NRP-1 in primary HNSCC tumors, the NRP-1 expression levels were assessed in a cohort of 172 HNSCC human samples selected from the archives of Pathology Unit of University “Federico II” of Naples, with a validated follow-up, by performing a tissue microarrays immunohistochemical analysis (TMA IHC). The study population features are reported in Table 3. We conducted a digital image analysis in order to quantify the tissue expression of NRP-1 protein. Using the QuPath software, we calculated the H-score of NRP-1 in both the oral squamous cell carcinomas (OSCC) and oropharyngeal squamous cell carcinomas (OPSCC) samples. Based on the threshold value calculated with the ROC curves, we grouped the samples into the two categories: NRP-1-LOW and NRP-1-HIGH. In the OSCC tumors, we observed low NRP-1 expression levels in 89/119 (74.8%) and high NRP-1 expression level in 30/119 (25.2%) cases. In the OPSCC, we classified as 20 out of 53 (37.7%) low NRP-1 samples and 33 out of 53 (62.3%) as high NRP-1. The NRP-1 cellular localization resulted in being both cytoplasmic and membranous, and both were evaluated for analysis (Table 4) (Figure 6). We analyzed the survival curves of both OSCC and OPSCC patients, stratifying the risk based on the expression of NRP-1 reported as high and low expression. We found a significant difference between the NRP-1-HIGH and NRP-1-LOW curves in both OSCC and OPSCC (*p* < 0.05). Of note, a high NRP-1 expression significantly correlated with a shorter overall survival rate in both the tumor subsites (Figure 7).

In our case series, out of 53 OPSCC cases, 7 were HPV positive and 46 HPV negative. One positive was NRP-1 HIGH, six were NRP-1 LOW. Out of the 46 HPV negative OPSCC, 32 were classified as NRP-1 HIGH and 14 as NRP-1 LOW. Contingency analysis showed an encouraging statistically significant frequency distribution of NRP-1 expression by HPV status in OPSCC (*p* = 0.004939). (Table 5).

The prognostic significance in OPSCC was also confirmed by the Neuropilin-1 mRNA values on the basis of the analysis carried out on the TCGA Head and Neck dataset, consisting of 315 OSCC and 79 OPSCC samples with valid NRP1 gene expression and follow-up data. NRP1 gene expression significantly stratified the risk in the OPSCC cohort. Interestingly, in 32/79 of the reported HPV positive cases, with better prognosis, as many as 30 are characterized by a low expression of the NRP1 gene. Data on OSCC cohort are unreliable since the analysis gave a false discovery rate of >50% (Table 6).

## 4. Discussion

Despite recent advances and the biological understanding of the head and neck cancers, patients outcomes have not substantially improved in recent years. Up to now, the prognosis of HNSCC patients remains mainly determined by the stage of the tumor at presentation, where the tumor size, the presence of lymph-node, and distant metastases, as well as persistent infection of high-risk HPVs, determined the stage. Concerning therapies, the standard of care for these patients consists of surgery, radiotherapy, chemotherapy, and recently, immunotherapy. However, the 5-year survival rate for HNSCC patients remains poor, accounting for 40–50% of mortality [1]. Concurrent radio- and chemotherapy may ameliorate survival and organ preservation, even if failing through the specific targeting of cancer cells results in high toxicity. Nevertheless, molecular-targeted therapies might potentially improve the outcome of the disease by targeting aberrant growth factor pathways specific to malignant cells rather than for all the rapidly proliferating cells without increasing the toxicity. The limited information available on the biology of HNSCC claims an urgent search for molecular prognostic and predictive biomarkers that might help this class of patients [30,31,32,33]. EGFR is one of the best candidates and is overexpressed in about 90% of HNSCC patients [34,35]. The use of the EGFR inhibitor, Cetuximab, in combination with radio and chemotherapy, represents the first targeted therapy scheme approved by the FDA as a standard of care for patients with recurrent or metastatic HNSCC [5,6,27,36]. NRP-1 as a co-receptor of the EGFR has been found widely expressed in a variety of human tumors [37,38,39,40,41]. Then, Neuropilin-1 represented an interesting candidate to investigate in HNSCC too. To this aim, we immunostained a tissue microarray-based case series of 218 HNSCCs with an anti-NRP-1 primary antibody. We obtained 172 valuable cores that we analyzed for the NRP-1 expression levels, correlating the results with the clinico-pathological features of the patients. High levels of NRP-1 expression significantly correlated with a shorter overall survival in both OSCC ad OPSCC diagnosed patients, letting us to envisage a potential prognostic role for this protein. Interestingly, the contingency analysis reported a statistically significant distribution of NRP1 tissue expression in HPV-positive and HPV-negative OPSCC cohorts from our studied case series since 6/7 HPV-positive cases were NRP1-LOW, 32/46 HPV-negative were NRP1-HIGH, and 14/46 HPV-negative were NRP1-LOW. Additionally, the TCGA data analysis also revealed that, in the OPSCC cohort, Neuropilin-1 mRNA values significantly correlated with prognosis. Indeed, data analysis of the TCGA OPSCC cohort (*n* = 79) indicated that, in 32 HPV-positive cases, as many as 30 showed a low expression of the NRP1 gene. Overall, our investigation suggests that the HPV-positive tumors mostly include the low NRP-1 expressing samples, while the HPV-negative tumors mainly comprise the high NRP-1 specimens. Thus, the prognostic role of NRP-1 is likely related to the HPV status and deserves further investigations.

Tumor cell proliferation and survival, angiogenesis, metastasis formation, and tumor immune escape include a series of mechanisms involving NRP-1 at different levels [14,37,38,39]. The ability to control multiple signaling pathways in different cell types may support the pleiotropic functions of NRP-1, sustaining the hypothesis that NRP-1 might represent a suitable target for cancer therapies [42]. It has been demonstrated that the upregulation of NRP-1 elicits adaptive resistance to oncogene-targeted therapies [43]. However, a role for NRP-1 in chemotherapy sensitivity has not been investigated yet. To address this point, by performing a stable NRP-1 depletion in the HNSCC cell lines HN6 and CAL33, carrying EGFR amplification and wild-type status, respectively, an increased sensitivity to chemotherapy was observed in both the HNSCC cells, which is in support of a role for NRP-1 in cisplatinum sensitivity. However, the HN13 cells, which carry a H773Y point mutation besides a EGFR amplification [29], did not exhibit an increased CDDP sensitivy upon NRP-1 silencing (Figure 5C). In primary tumors, the failure to standard therapies, upon the development of resistance, is often associated with the activation of side signaling pathways. Indeed, CDDP-induced cytotoxic stress activates several signaling pathways that affect cell growth and survival, cell cycle, DNA repair, and drug transport. One of these pathways is represented by the EGFR pathway [44]. By the investigations performed in this study, NRP-1 resulted in being able to sustain the cisplatin-induced EGFR activation since it was able to enhance the EGFR signaling upon ligand stimulation, as already reported in additional cellular models [45]. Of note, we observed that the NRP-1 depletion severely impaired the cisplatin-induced EGFR phosphorylation. Differently from the common rapid and transient EGFR activation in response to stimulation with its physiological ligands, CDDP-mediated EGFR activation occurred several hours after the beginning of the treatment. The delayed EGFR activation, approximately 6 h after CDDP treatment, is consistent with the chemotherapeutic drug mechanism of action, which acts by the formation of DNA adduct. Then, we hypothesize that NRP-1 may participate in the control of EGFR activation with a different mechanism with respect to the ligand-dependent one. The Neuropilin-1 depletion in the HN13 cells did not affect EGFR activation, probably on account of the H773Y mutation (Figure 4B). This observation suggested that NRP-1 might serve as a EGFR co-receptor, mainly acting through the residue Y1068.

It has been reported that CDDP may induce EGFR phosphorylation in a ligand-independent manner by involving additional kinases [44]. Therefore, NRP-1 might work as an additional player in the regulation of EGFR signaling by also acting in synergy with different kinases, such as Src [46].

## 5. Conclusions

On the basis of the data we have reported and in a translational perspective, NRP-1-interfering molecules, such as nanobodies or small molecules interacting with the extracellular domain [47], might be used in combined therapeutic regimens for HNSCC. In conclusion, we believe that the data presented here suggest a prognostic role for NRP-1 in HNSCC patients. Furthermore, we provided some observations about the NRP-1 contribution to the cisplatin sensitivity in HNSCC tumors both in in vitro experiments and in the clinical setting; the latter deserves further investigations, such as in a clinical trial. Mechanisms of chemoresistance may be mediated by NRP-1 in HPV-negative tumors, which are harder to treat due to chemoresistance to the only targeted therapy involving EGFR inhibition described so far [48].

We extended the repertoire of signaling in which NRP-1 is involved showing, for the first time, that NRP-1 controls the cisplatin-induced EGFR signaling at least through the residue Y1068.

This observation opens the way to further investigations in order to understand the functional impact of NRP-1 on the control of the EGFR pathway, activated in response to chemotherapy, and the effect of NRP-1 targeting as a novel strategy for personalized treatments in HNSCC.

## Figures and Tables

**Table 1:** EGFR mutational status in CAL33, HN6, and HN13 HNSCC cells.

Cell Lines	EGFR Mutational Status	References
CAL33	wild type	[27]
HN6	amplified	[28]
HN13	mutated (p.H773Y) and amplified	[29]

**Table 2:** 50% inhibitory concentration values (IC50) calculated for HN6, CAL33, and HN13 cell lines in a cell viability assay of NRP-1 silenced- compared to the control-cells upon cisplatin treatment.

Cisplatin IC 50 (μM)		
	**shNRP-1**	**pLKO**
**HN6**	2.5	5.5
**CAL33**	5	12
**HN13**	1.9	2

**Table 3:** Clinico-pathological features of the study population (NOP: Non-Oropharyngeal; OP: Oropharyngeal; A&W: Alive and Well; DOD: Dead of Disease).

		N	%
GENDER	F	76	44%
	M	96	56%
	Total	172	100%
AGE	Mean	63.67	
	Median	64.00	
	Minimum	33	
	Maximum	89	
SITE	NOP	119	69%
	OP	53	31%
	Total	172	100%
GRADE	G1	6	3%
	G1/G2	8	5%
	G2	47	27%
	G2/G3	18	10%
	G3	76	44%
	missing	17	10%
	Total	172	100%
STAGE AJCC VIIIed.	I	19	11%
	II	38	22%
	III	20	12%
	IVA	78	45%
	Missing	17	10%
	Total	172	100%
F-UP	A&W	93	54%
	DOD	79	46%
	Total	172	100%
F-UP (months)	Mean	29.36	
	Median	18.00	
	Minimum	1	
	Maximum	157	

**Table 4:** The crosstabulation of tumor site by tumor Neuropilin-1 H-score groups count.

			Tumor Neuropilin-1 H-Score		
			**HIGH**	**LOW**	**Total**
SITE	OSCC	Count	30	89	119
		% within SITE	25.2%	74.8%	100%
	OPSCC	Count	33	20	53
		% within SITE	62.3%	37.7%	100%
	Total	Count	63	109	172
		% within SITE	36.6%	63.4%	100%

**Table 5:** Contingency table of NRP-1 expression by HPV positivity in OPSCC tumor samples (total *n* = 53). The chi square statistic is 7.9016, and the *p*-value is 0.004939. Significance at <0.05.

		HPV	
		**POS**	**NEG**
**NRP-1**	**HIGH**	1	32
	**LOW**	6	14

**Table 6:** Analysis of NRP-1 gene expression in the TCGA Head and Neck dataset. The best *p* value cutoff for thresholding is shown. Significance at <0.05. (HR: Hazard Ratio; FDR: False Discovery Rate).

	Site (*n* of Cases with Valid F-UP)	
	**OSCC (** ***n*** ** = 315)**	**OPSCC (** ***n*** ** = 79)**
**Best cutoff**	10.45	10.53
**Logrank ** ***p*** ** value**	0.029	0.00023
**HR**	0.7 (0.5–0.97)	4.31 (1.85–10.04 )
**FDR**	>50%	1%

## Data Availability

TCGA-HNSC dataset is available at https://portal.gdc.cancer.gov (accessed on 14 July 2021); statistical tools for survival analysis are available at kmplot.com (accessed on 14 July 2021).

## Supplementary Materials

The following are available online at https://www.mdpi.com/article/10.3390/cancers13153822/s1, Figure S1: Original Western Blots: the original Wstern Blots are available as Supplementary Materials.

## Abbreviations

The following abbreviations are used in this manuscript:

HNSCC	Head and Neck Squamous Cell Carcinoma
OSCC	Oral Squamous Cell Carcinoma
OPSCC	Oropharyngeal Squamous Cell Carcinoma
NRP-1	Neuropilin1
MAPK	Map Kinase
CDDP	Cisplatin
EGF	Epidermal Growth Factor
HPV	Human Papilloma Virus
