# Supplementary figures and images for: Neuropilin-1 Expression Associates with Poor Prognosis in HNSCC and Elicits EGFR Activation upon CDDP-Induced Cytotoxic Stress
Source: Cancers (Basel). 2021 Jul 29;13(15):3822. doi: 10.3390/cancers13153822 (PMC8345038; doi:10.3390/cancers13153822)

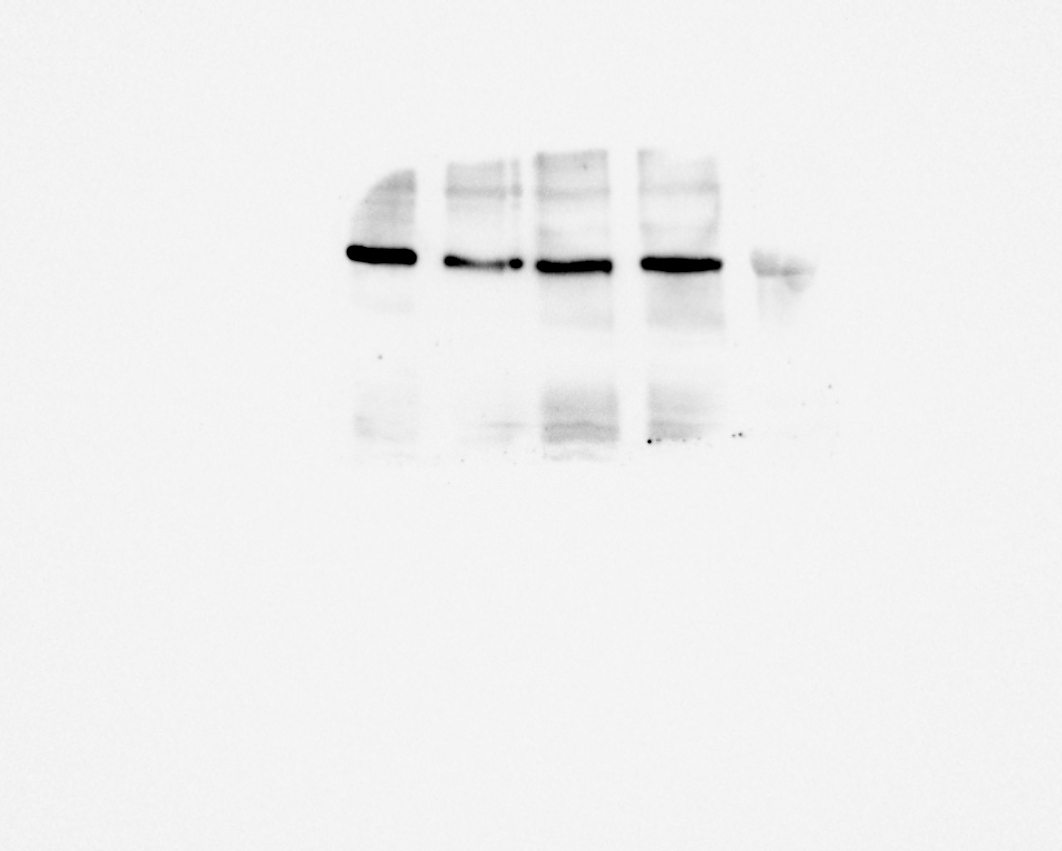

Supplement: Supplementary file 1 [file cancers-13-03822-s001.zip › Supplementary FIgure S1/Cal33_tubulin.jpg]

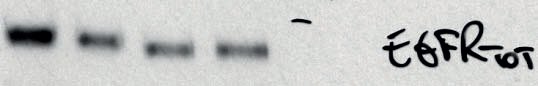

Supplement: Supplementary file 1 [file cancers-13-03822-s001.zip › Supplementary FIgure S1/Clipboard-1.jpg]

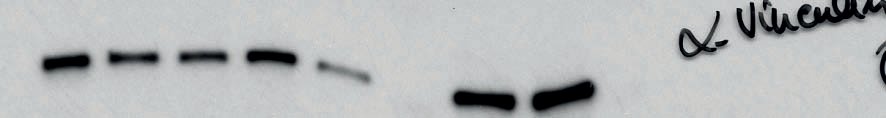

Supplement: Supplementary file 1 [file cancers-13-03822-s001.zip › Supplementary FIgure S1/Clipboard-2.jpg]

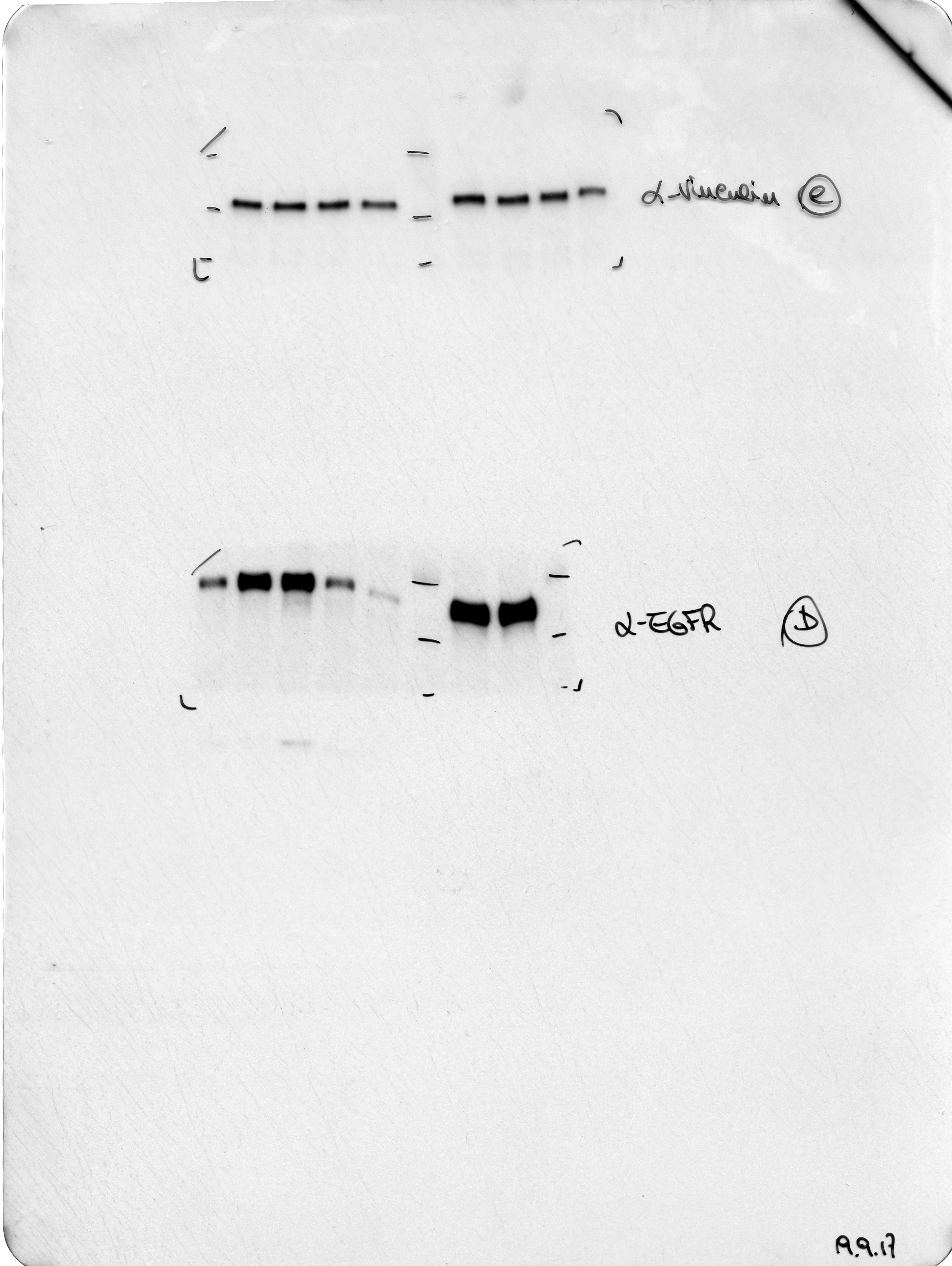

Supplement: Supplementary file 1 [file cancers-13-03822-s001.zip › Supplementary FIgure S1/Clipboard-4.jpg]

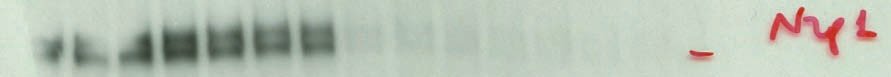

Supplement: Supplementary file 1 [file cancers-13-03822-s001.zip › Supplementary FIgure S1/Clipboard-6.jpg]

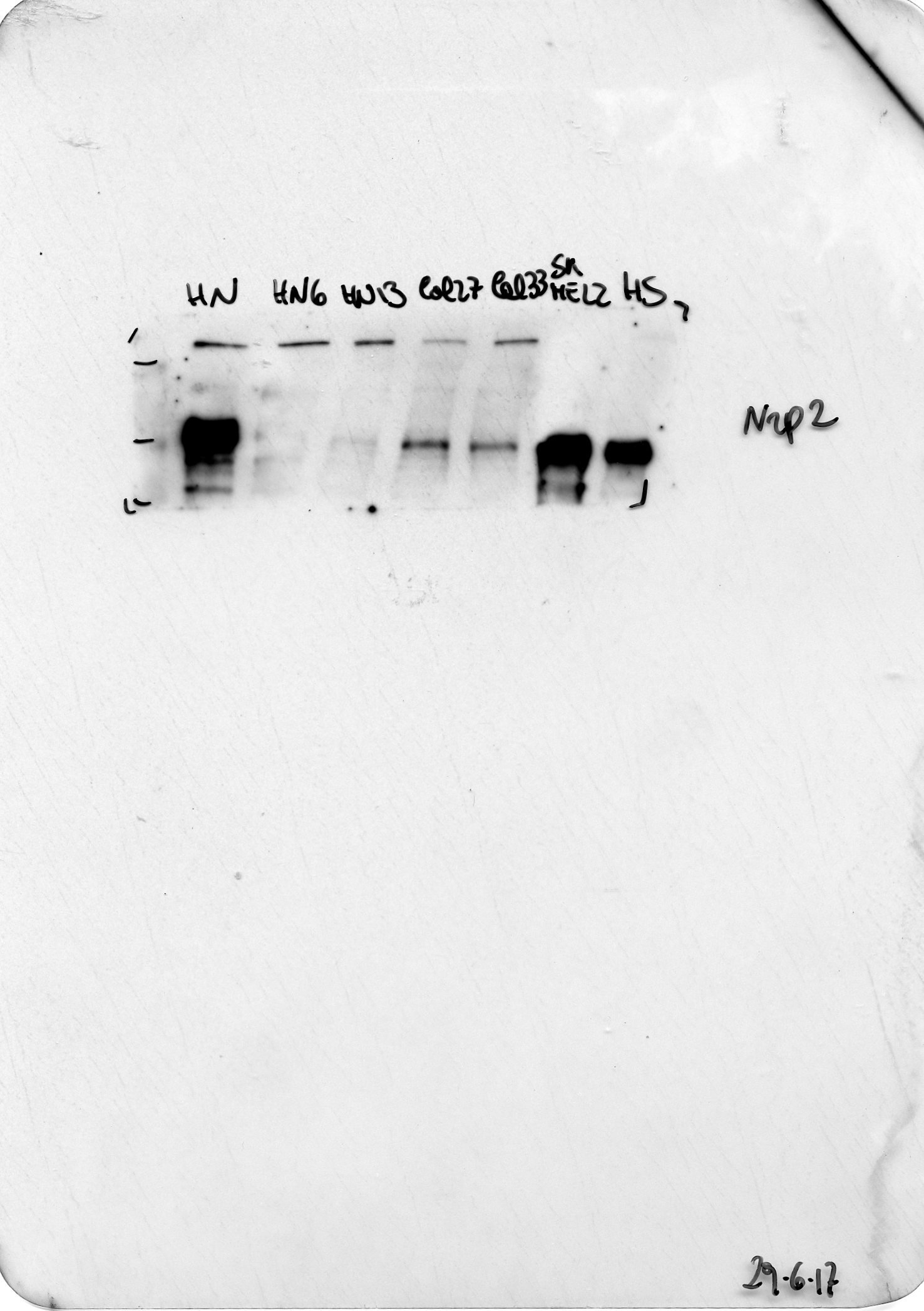

Supplement: Supplementary file 1 [file cancers-13-03822-s001.zip › Supplementary FIgure S1/Clipboard.jpg]

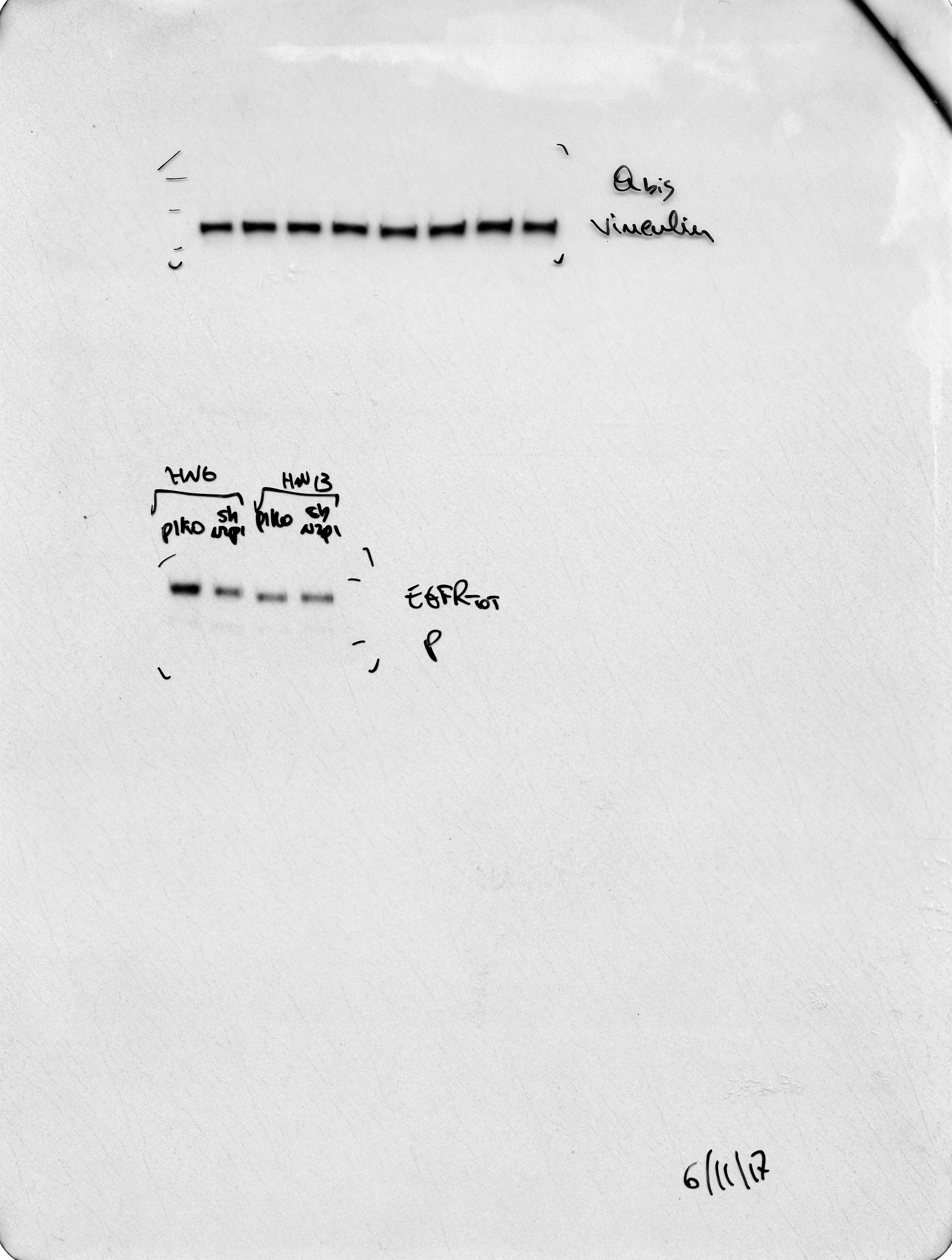

Supplement: Supplementary file 1 [file cancers-13-03822-s001.zip › Supplementary FIgure S1/Fig.2B_EGFR.jpg]

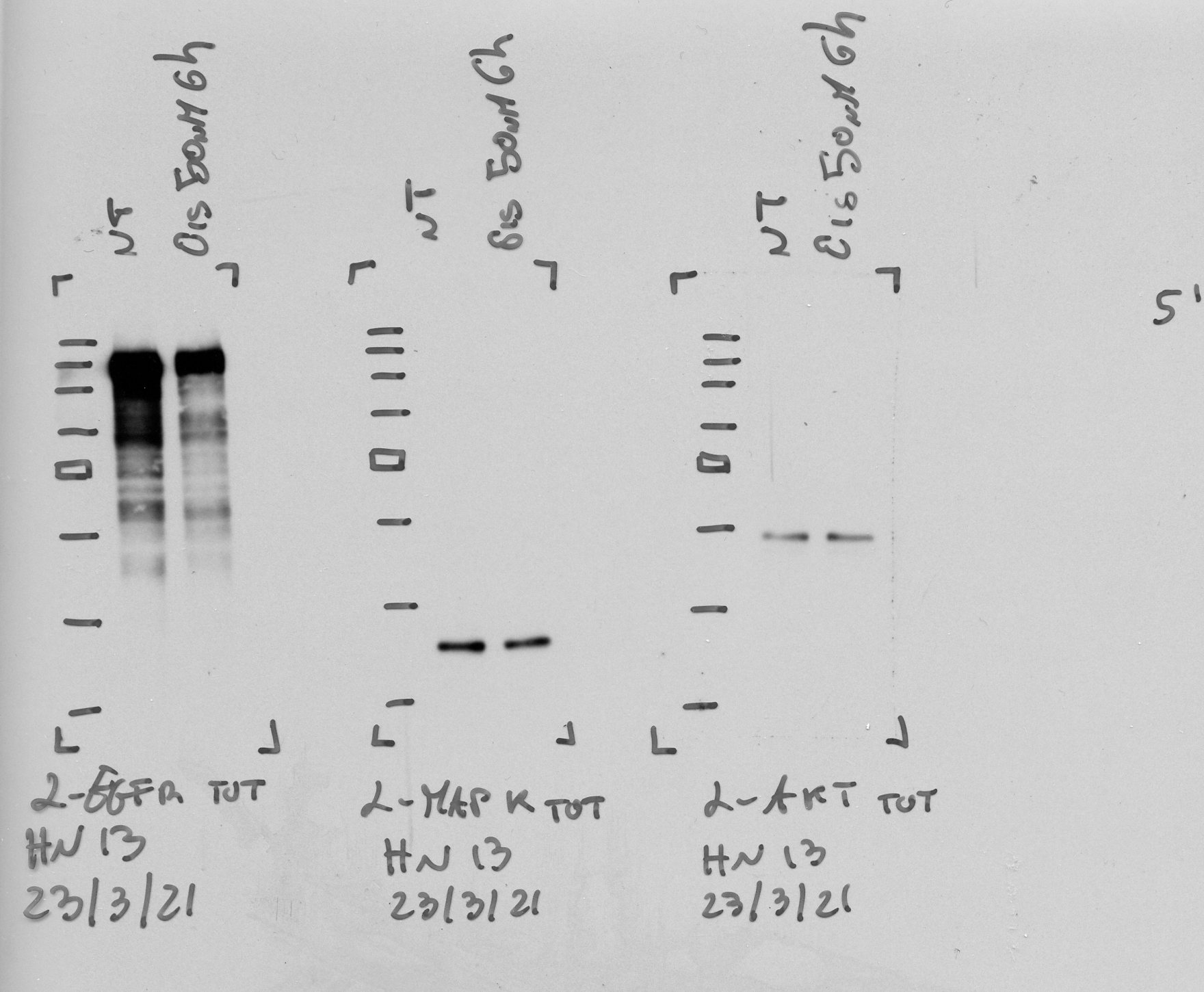

Supplement: Supplementary file 1 [file cancers-13-03822-s001.zip › Supplementary FIgure S1/img241.jpg]

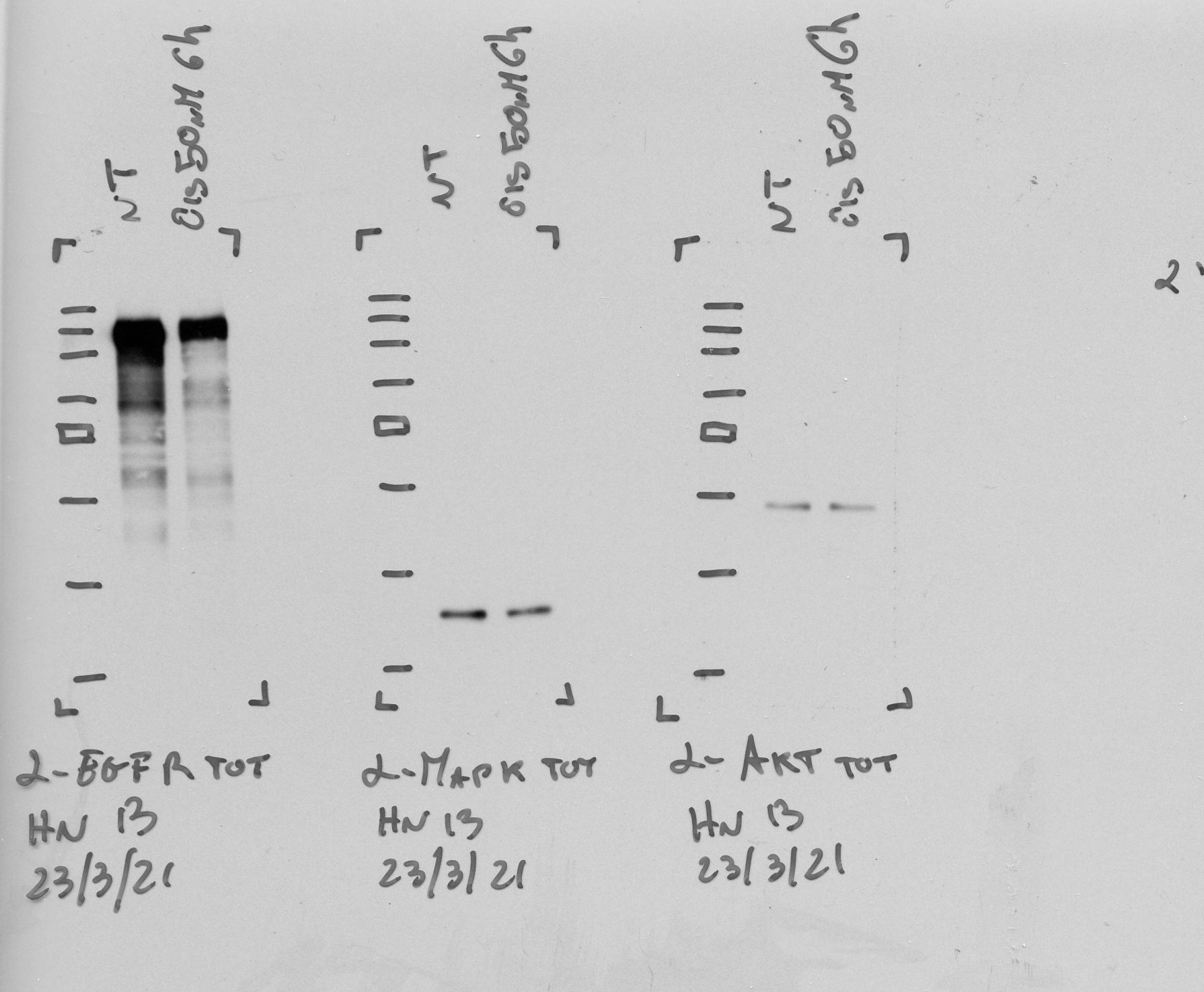

Supplement: Supplementary file 1 [file cancers-13-03822-s001.zip › Supplementary FIgure S1/img242.jpg]

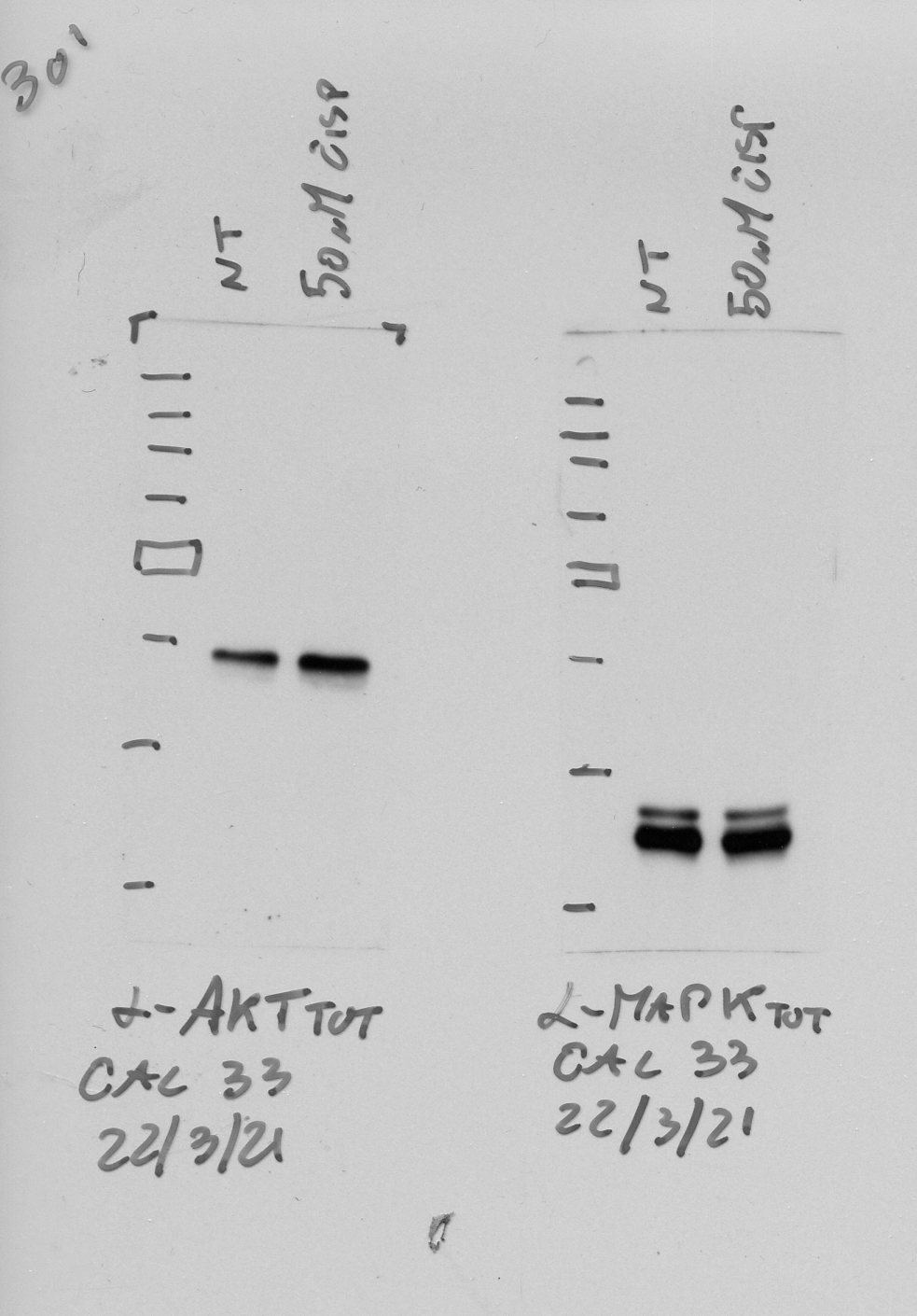

Supplement: Supplementary file 1 [file cancers-13-03822-s001.zip › Supplementary FIgure S1/img247.jpg]

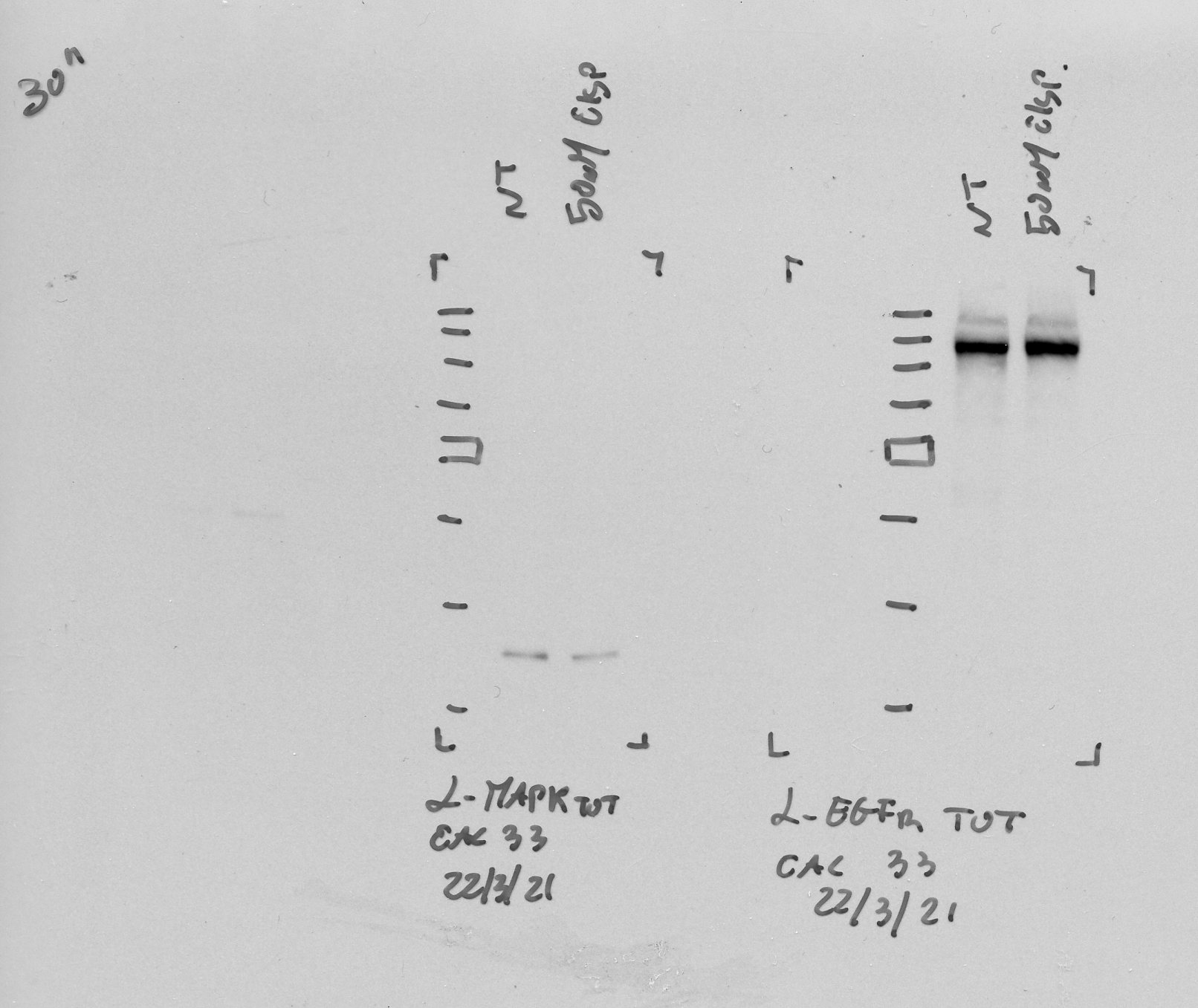

Supplement: Supplementary file 1 [file cancers-13-03822-s001.zip › Supplementary FIgure S1/img249.jpg]

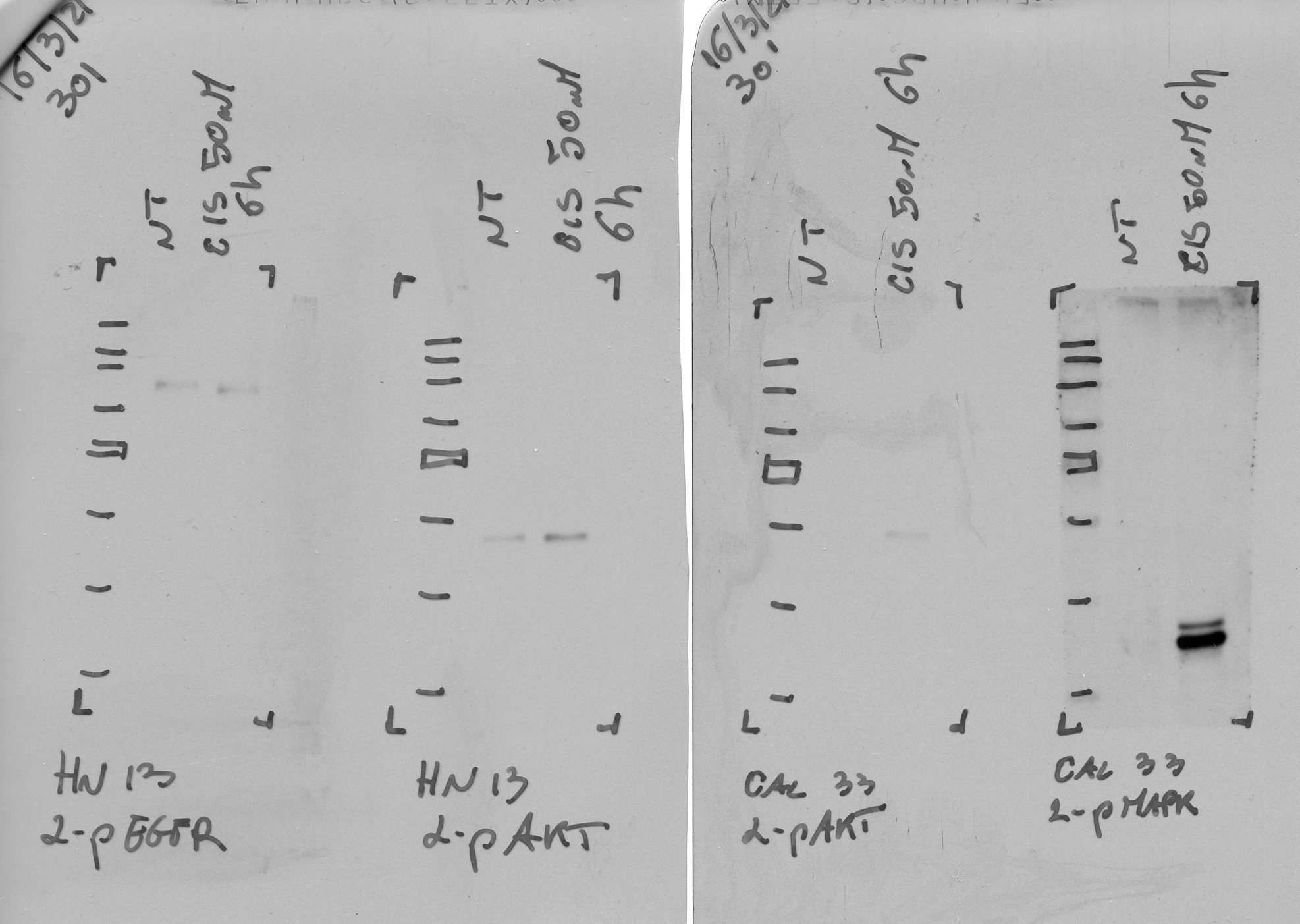

Supplement: Supplementary file 1 [file cancers-13-03822-s001.zip › Supplementary FIgure S1/img250.jpg]

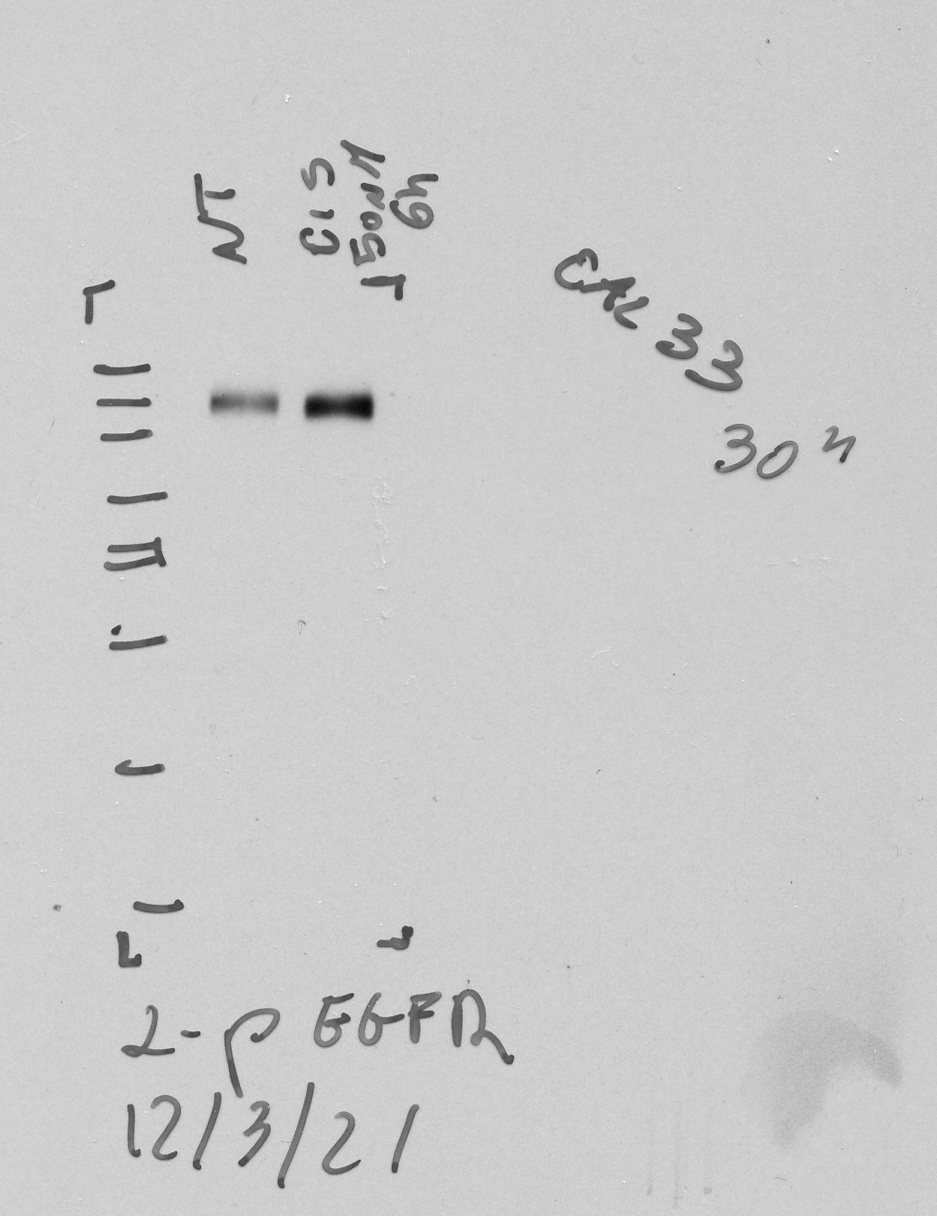

Supplement: Supplementary file 1 [file cancers-13-03822-s001.zip › Supplementary FIgure S1/img252.jpg]

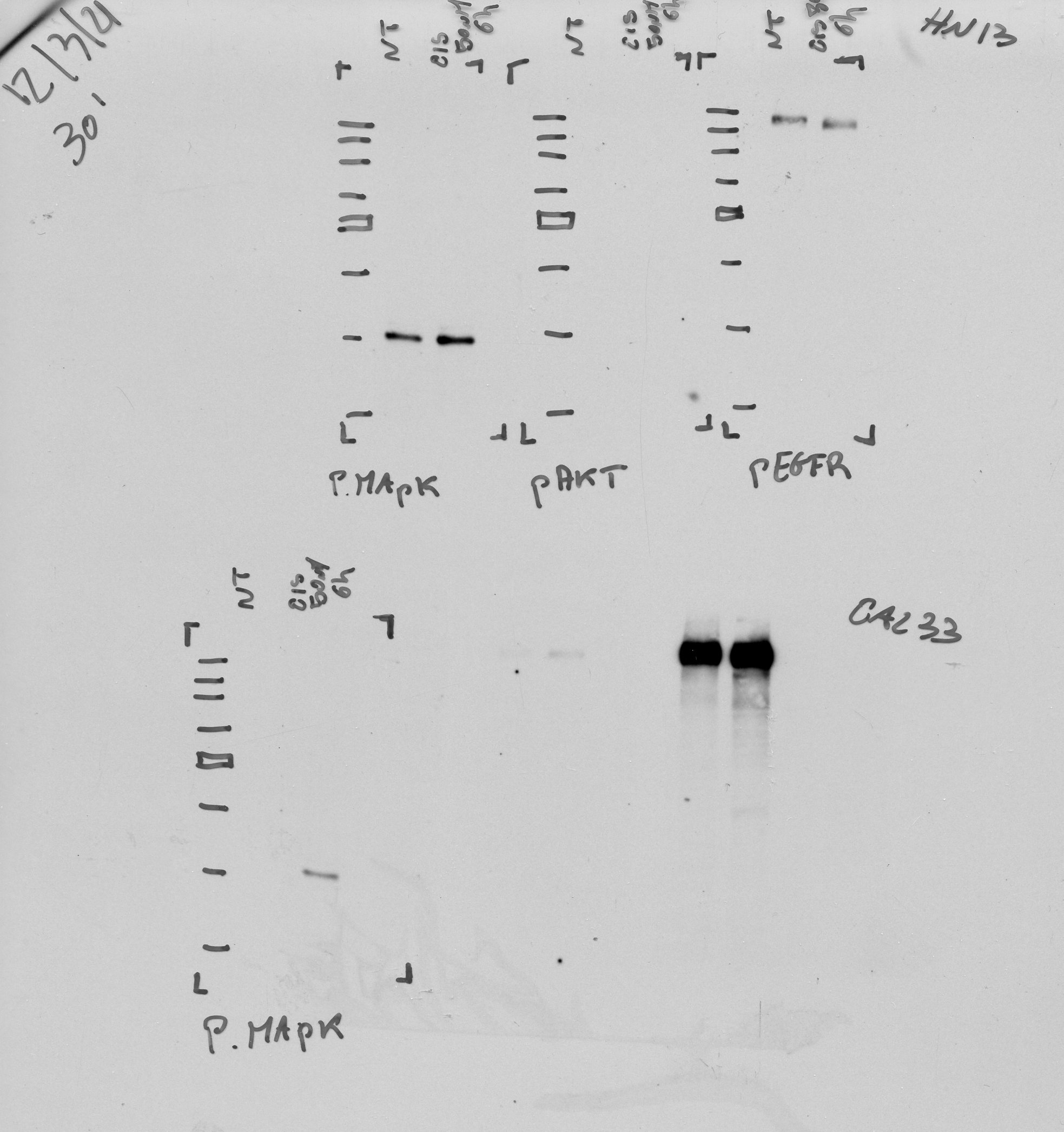

Supplement: Supplementary file 1 [file cancers-13-03822-s001.zip › Supplementary FIgure S1/img253.jpg]

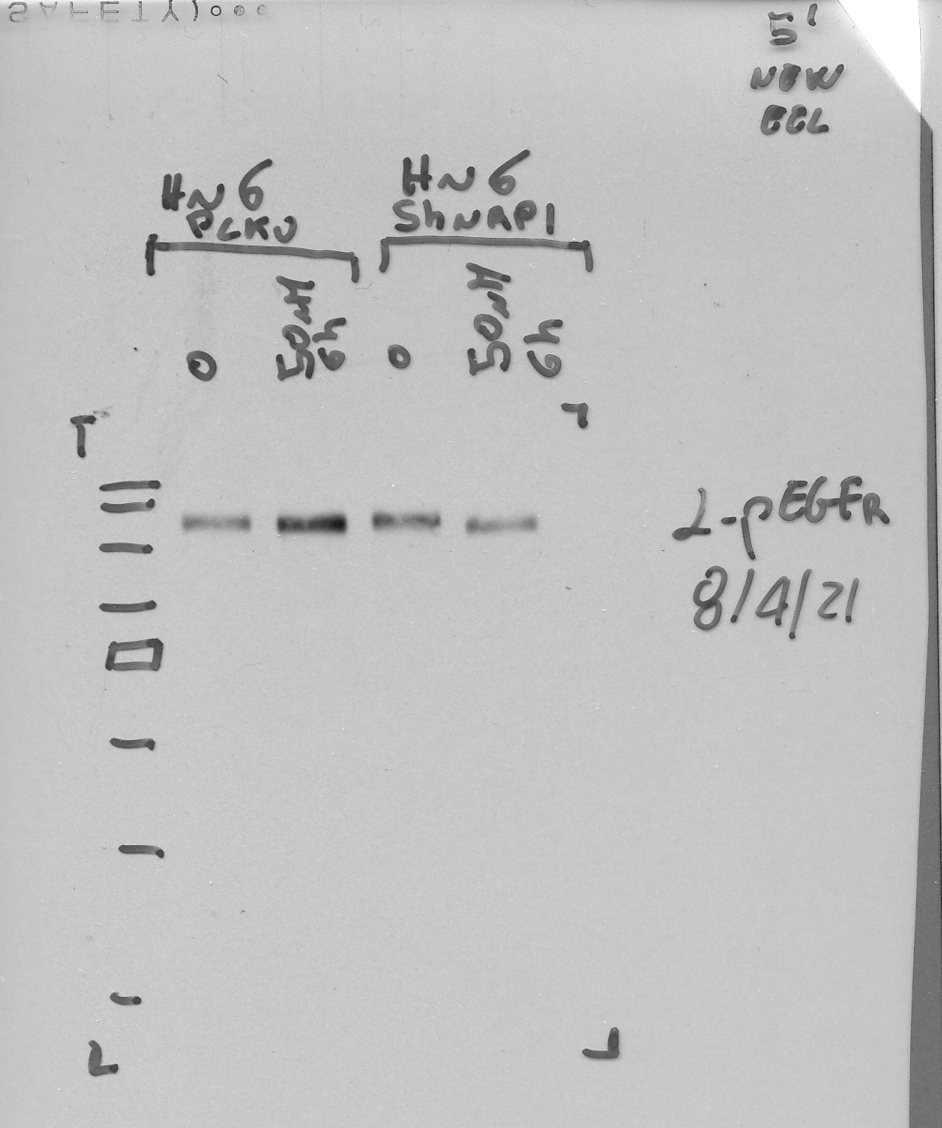

Supplement: Supplementary file 1 [file cancers-13-03822-s001.zip › Supplementary FIgure S1/img272.jpg]

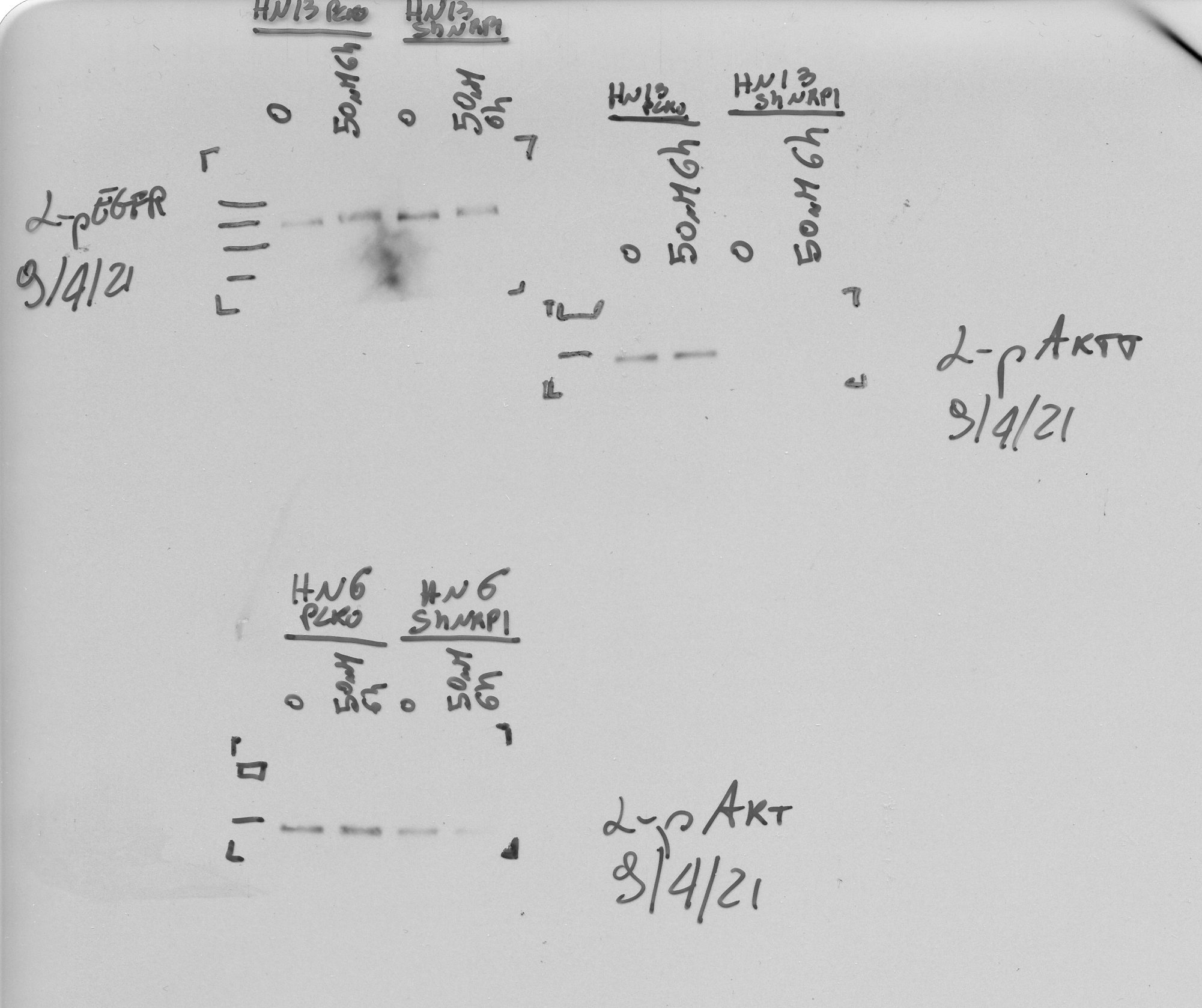

Supplement: Supplementary file 1 [file cancers-13-03822-s001.zip › Supplementary FIgure S1/img274.jpg]

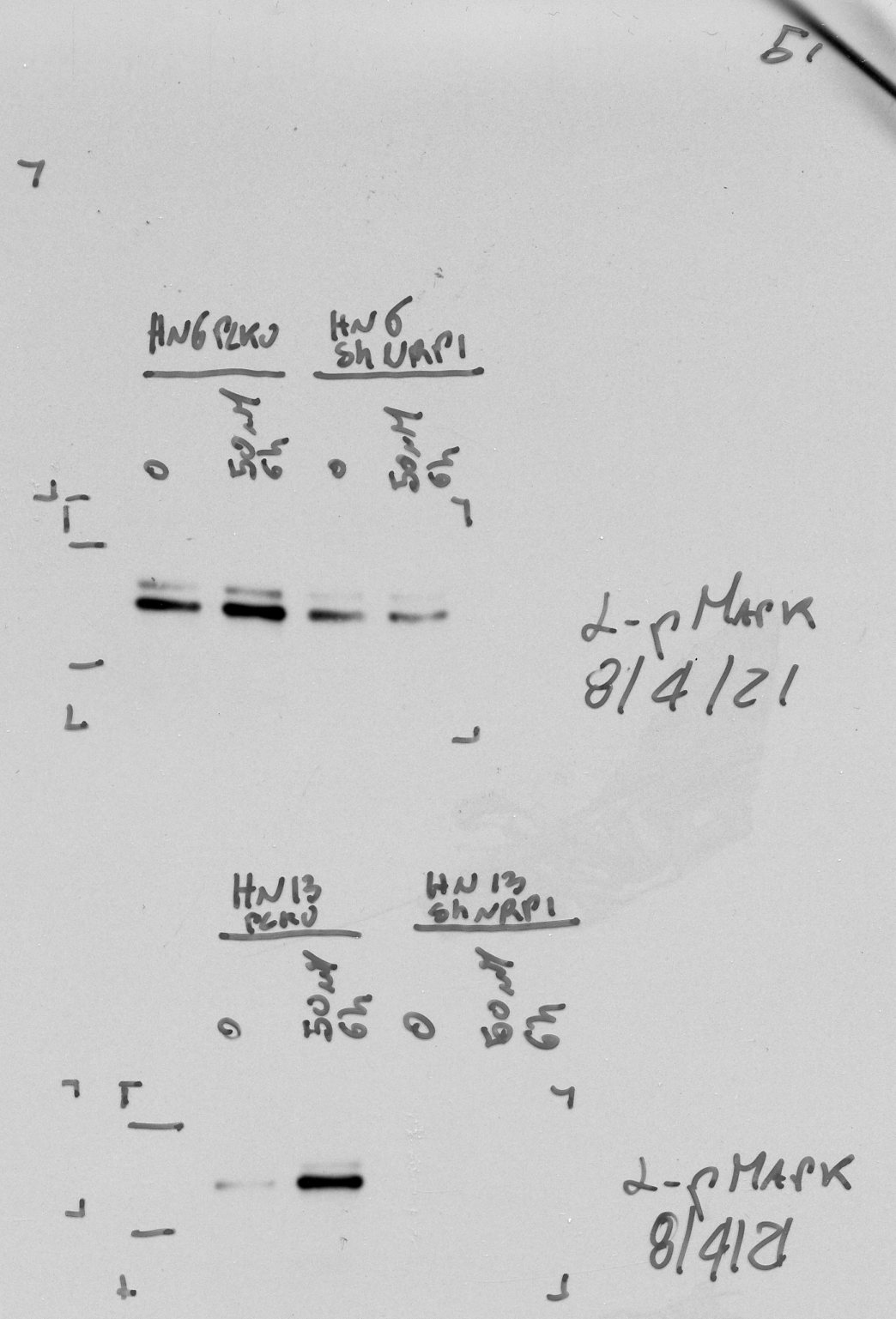

Supplement: Supplementary file 1 [file cancers-13-03822-s001.zip › Supplementary FIgure S1/img275.jpg]

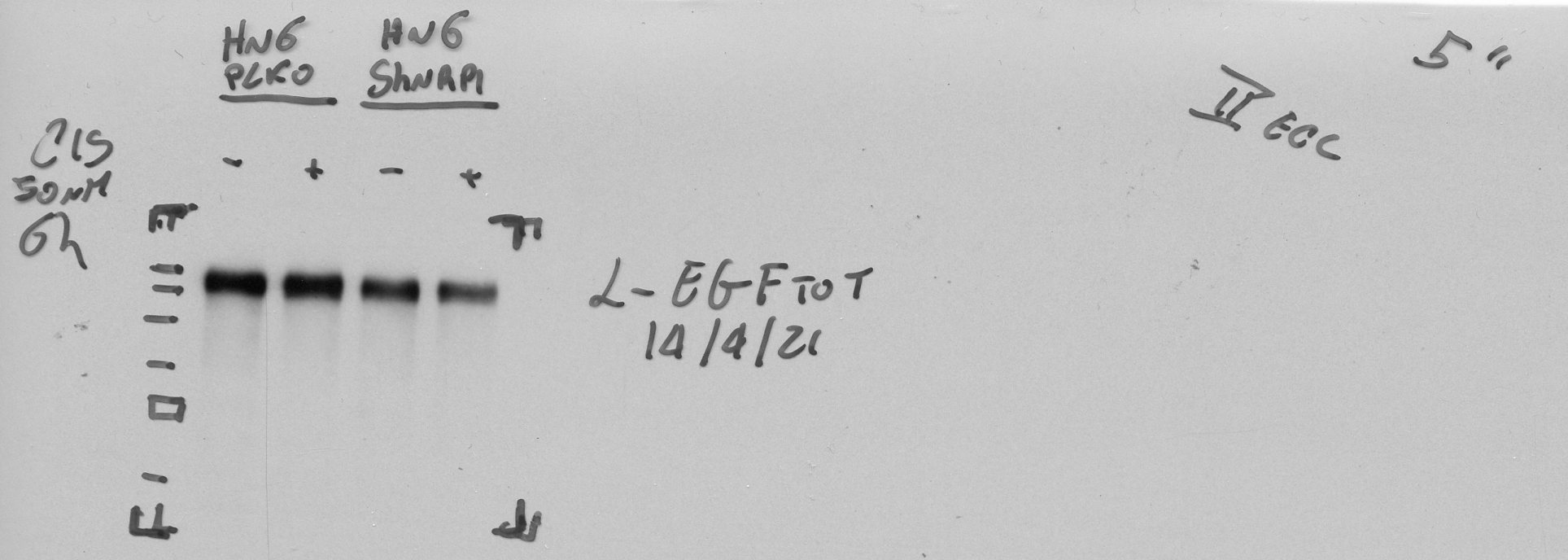

Supplement: Supplementary file 1 [file cancers-13-03822-s001.zip › Supplementary FIgure S1/img279.jpg]

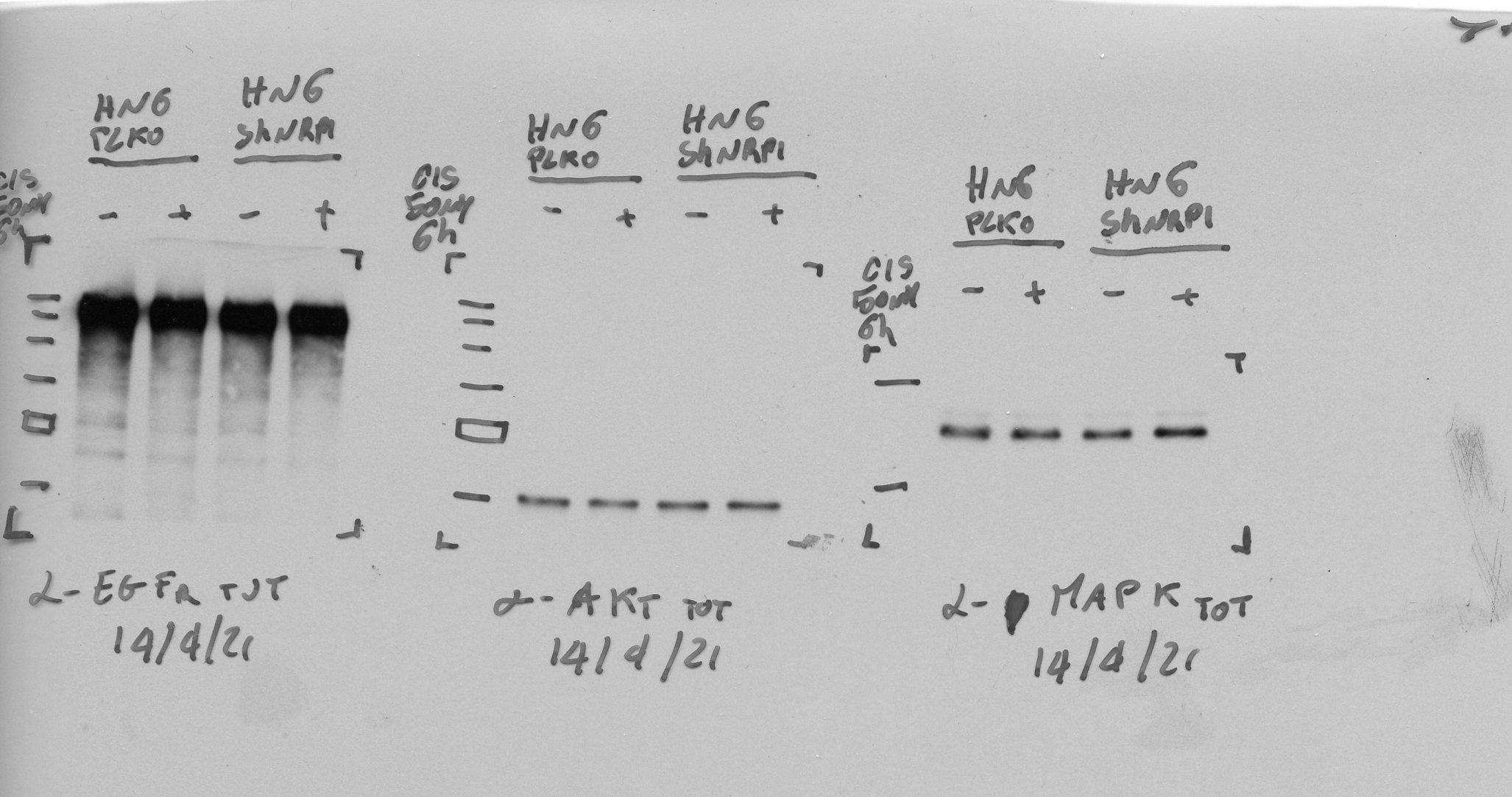

Supplement: Supplementary file 1 [file cancers-13-03822-s001.zip › Supplementary FIgure S1/img283.jpg]

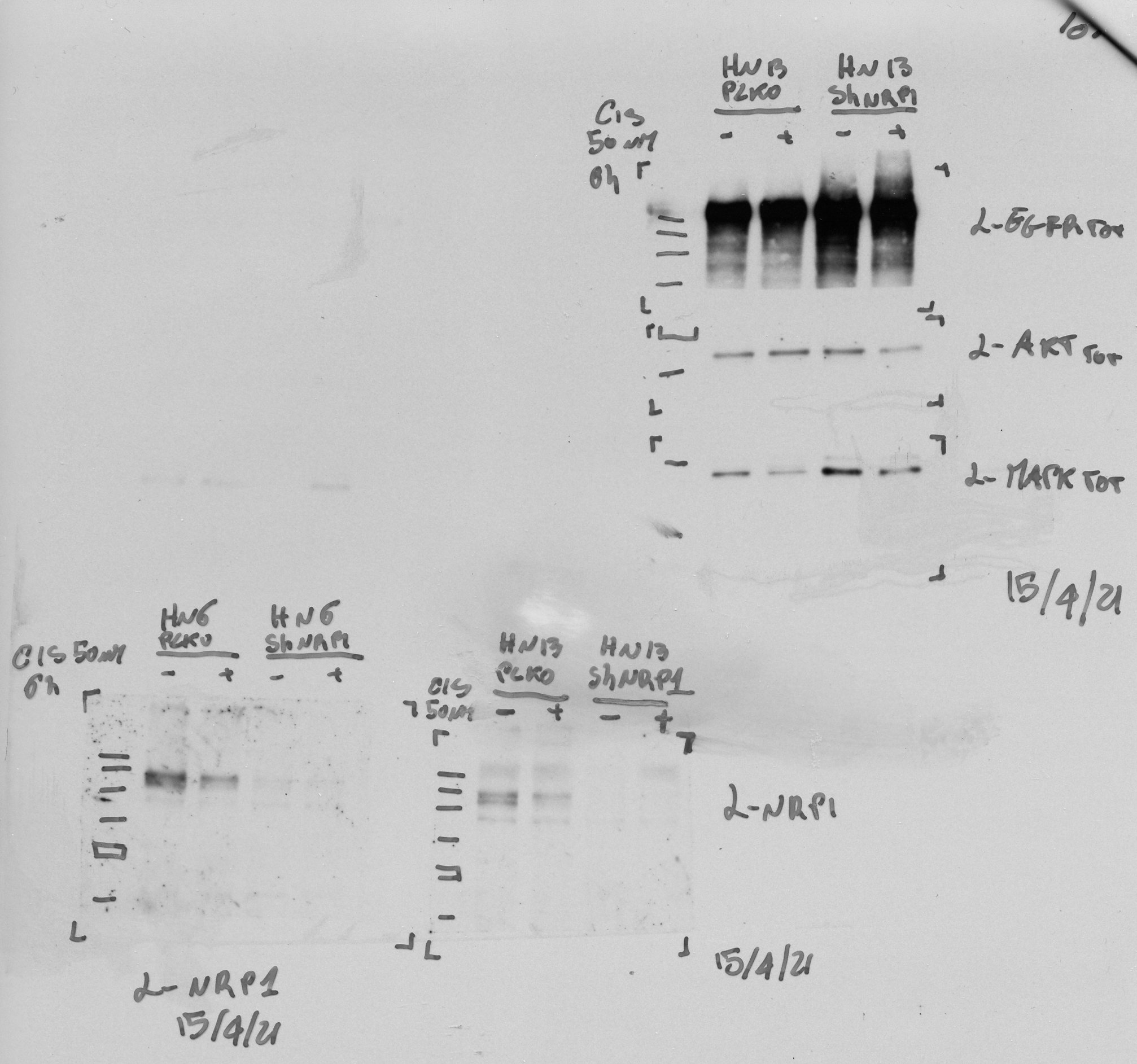

Supplement: Supplementary file 1 [file cancers-13-03822-s001.zip › Supplementary FIgure S1/img293.jpg]

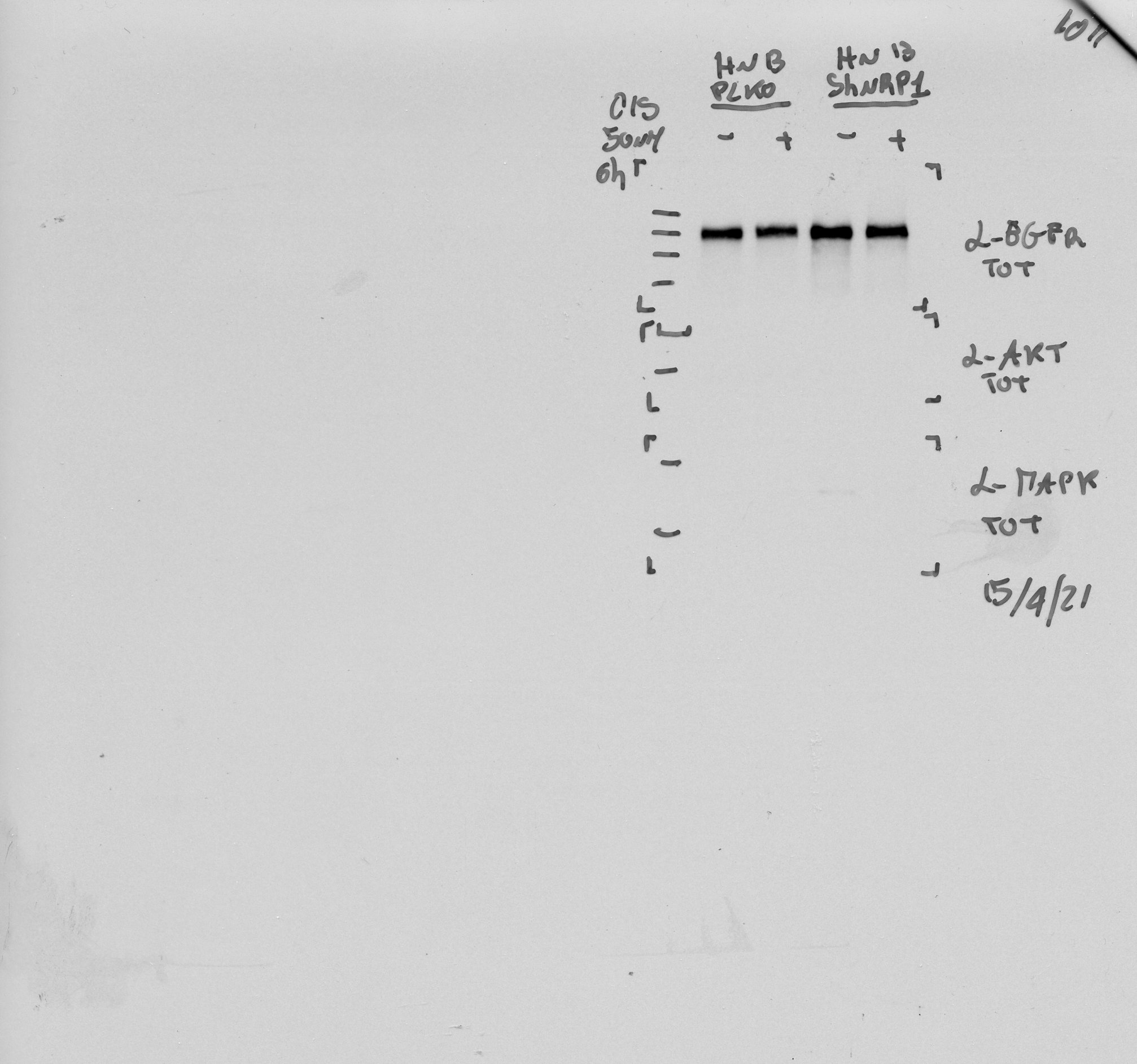

Supplement: Supplementary file 1 [file cancers-13-03822-s001.zip › Supplementary FIgure S1/img296.jpg]

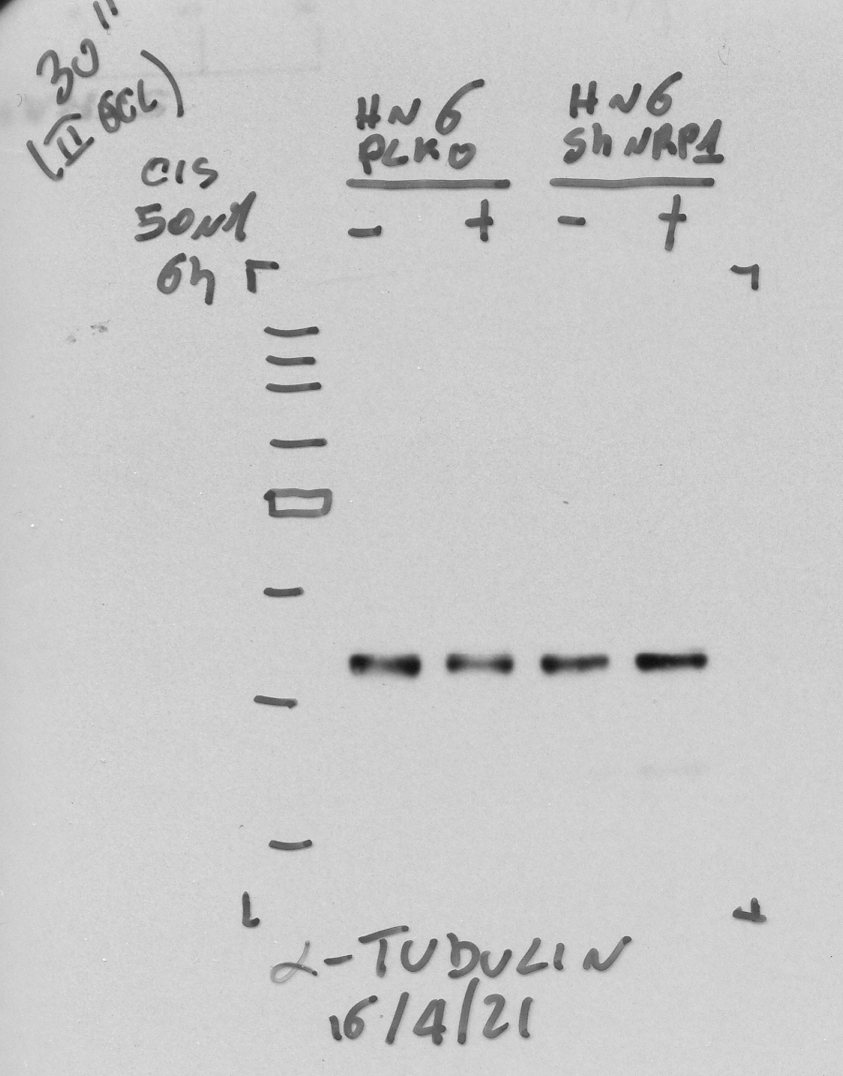

Supplement: Supplementary file 1 [file cancers-13-03822-s001.zip › Supplementary FIgure S1/img299-.jpg]

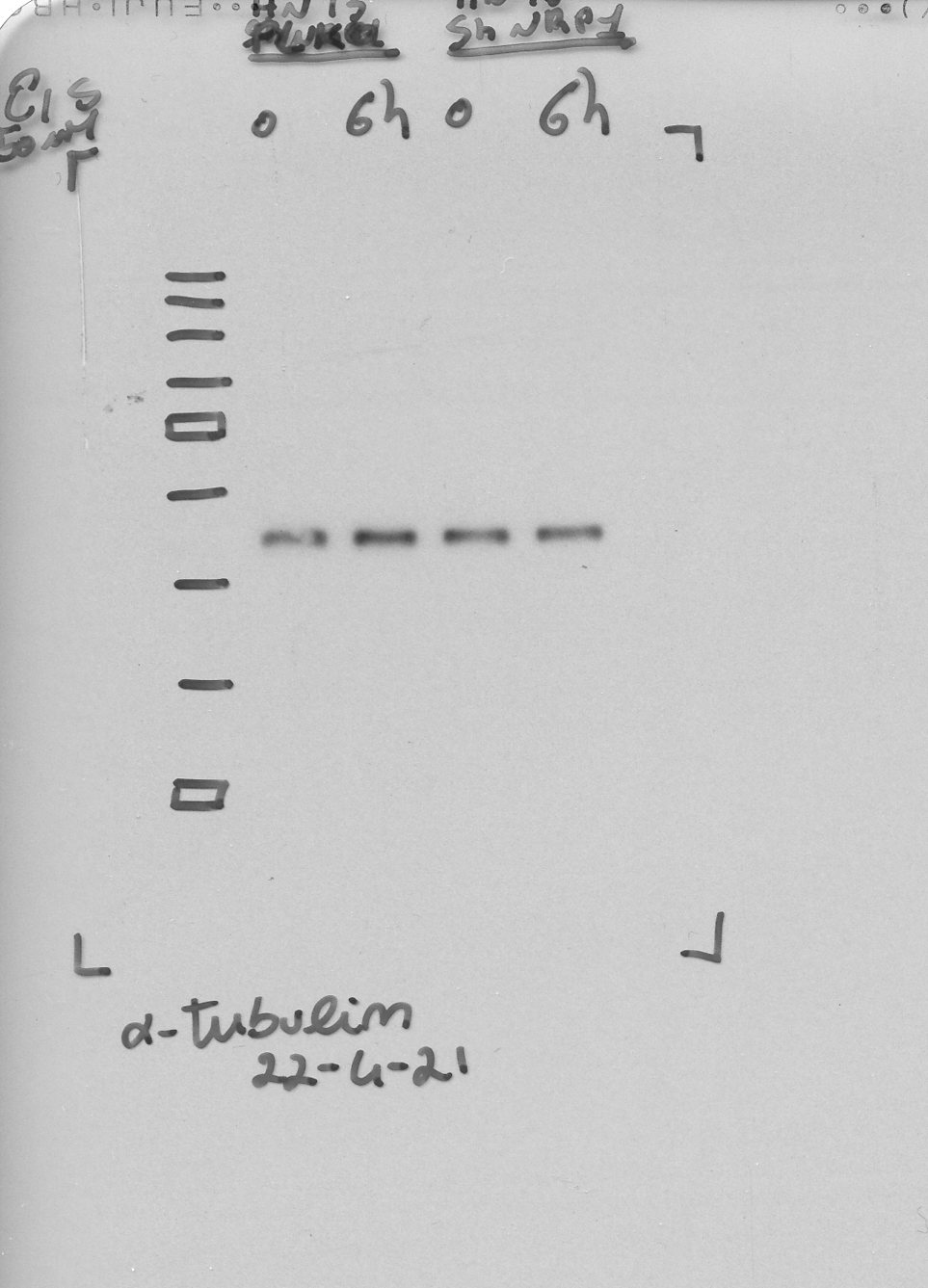

Supplement: Supplementary file 1 [file cancers-13-03822-s001.zip › Supplementary FIgure S1/img301.jpg]

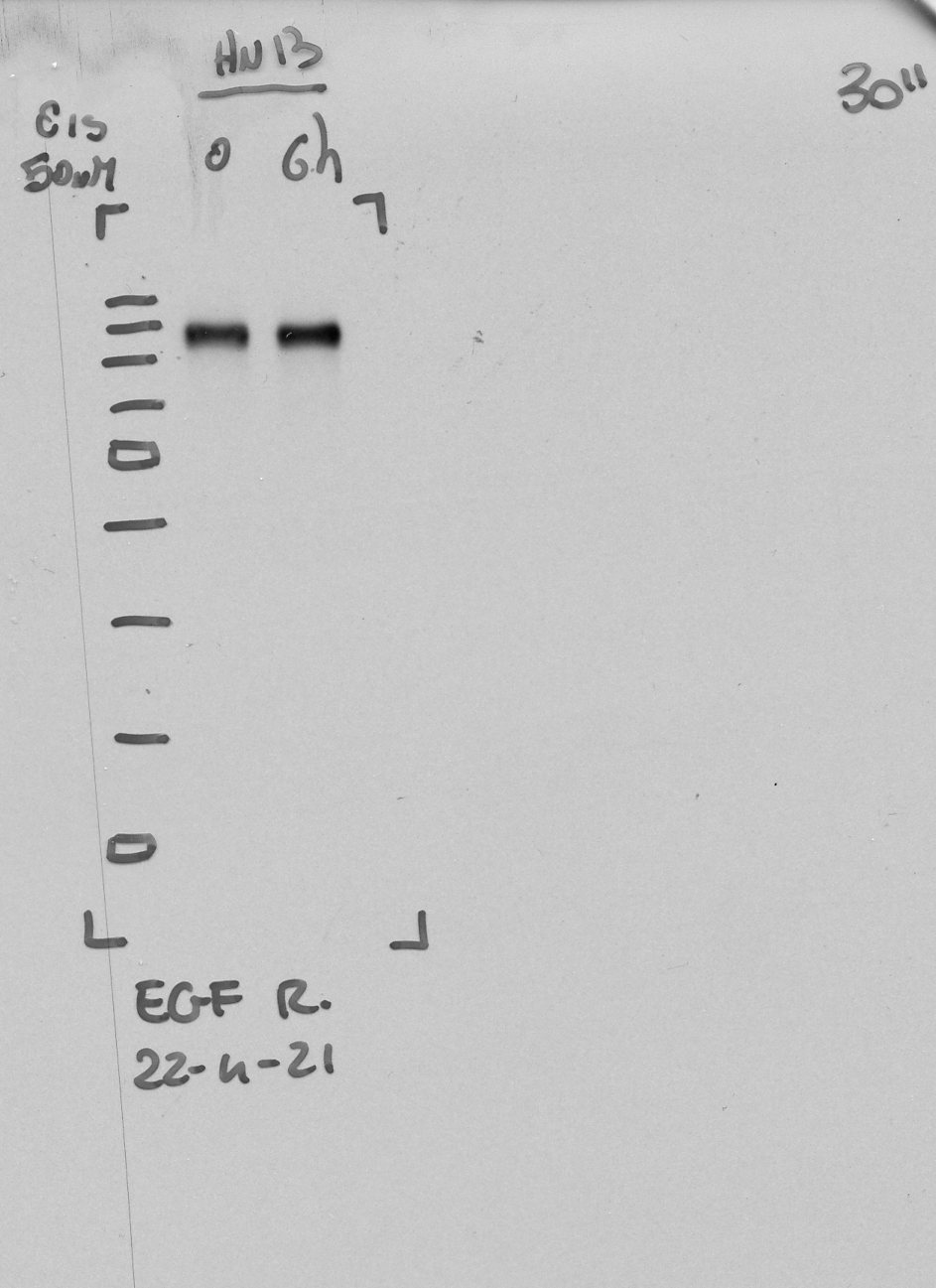

Supplement: Supplementary file 1 [file cancers-13-03822-s001.zip › Supplementary FIgure S1/img305.jpg]

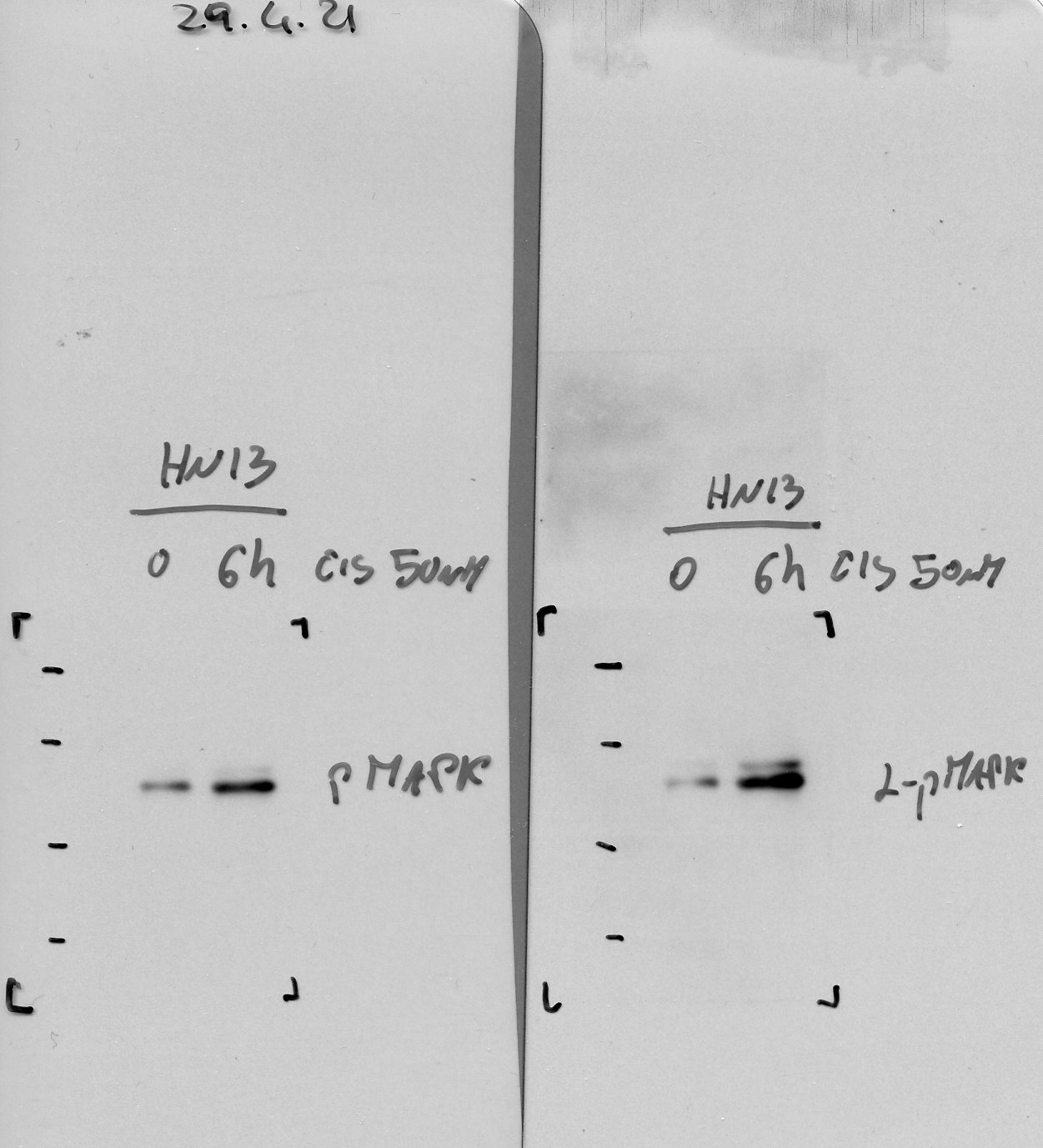

Supplement: Supplementary file 1 [file cancers-13-03822-s001.zip › Supplementary FIgure S1/img325.jpg]

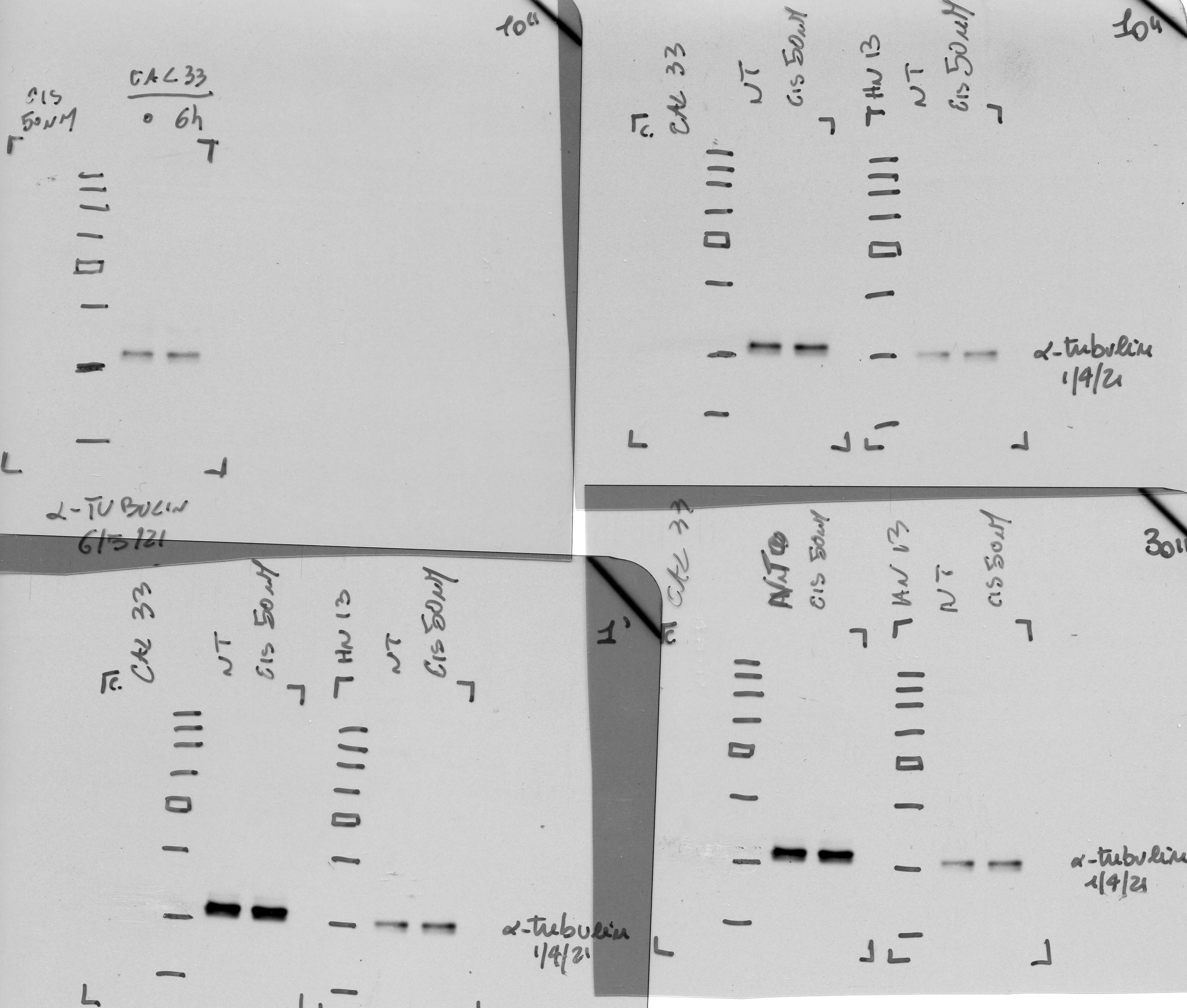

Supplement: Supplementary file 1 [file cancers-13-03822-s001.zip › Supplementary FIgure S1/img339.jpg]

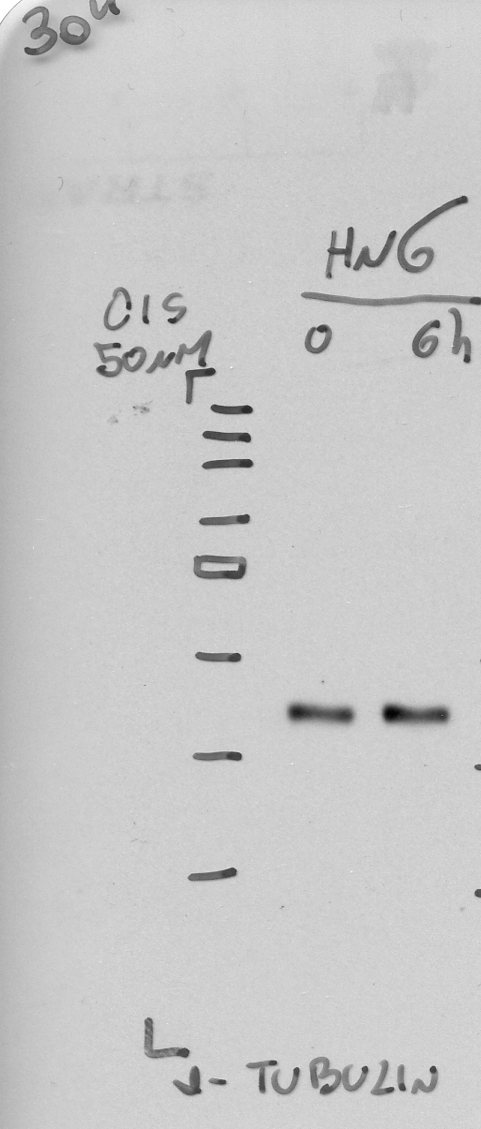

Supplement: Supplementary file 1 [file cancers-13-03822-s001.zip › Supplementary FIgure S1/img340.jpg]

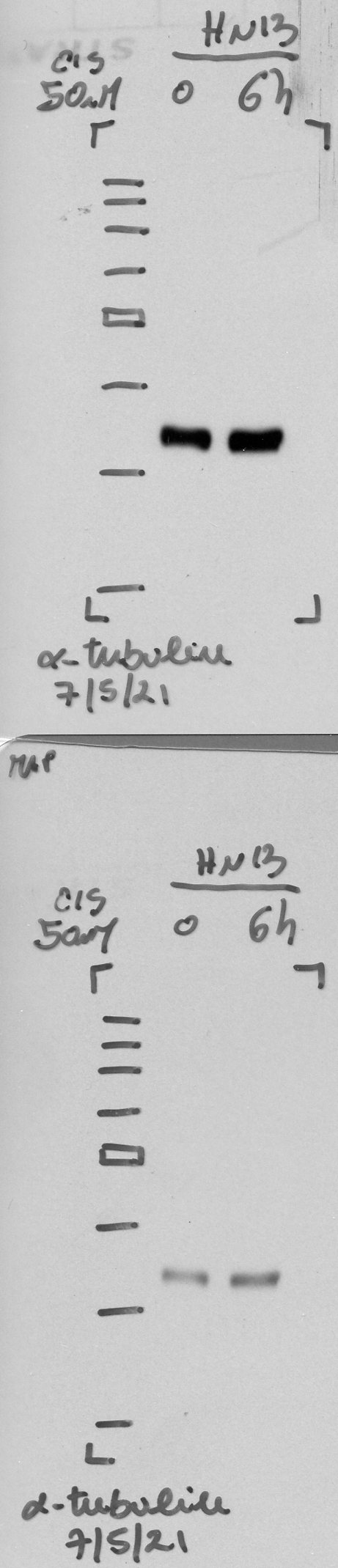

Supplement: Supplementary file 1 [file cancers-13-03822-s001.zip › Supplementary FIgure S1/img346.jpg]

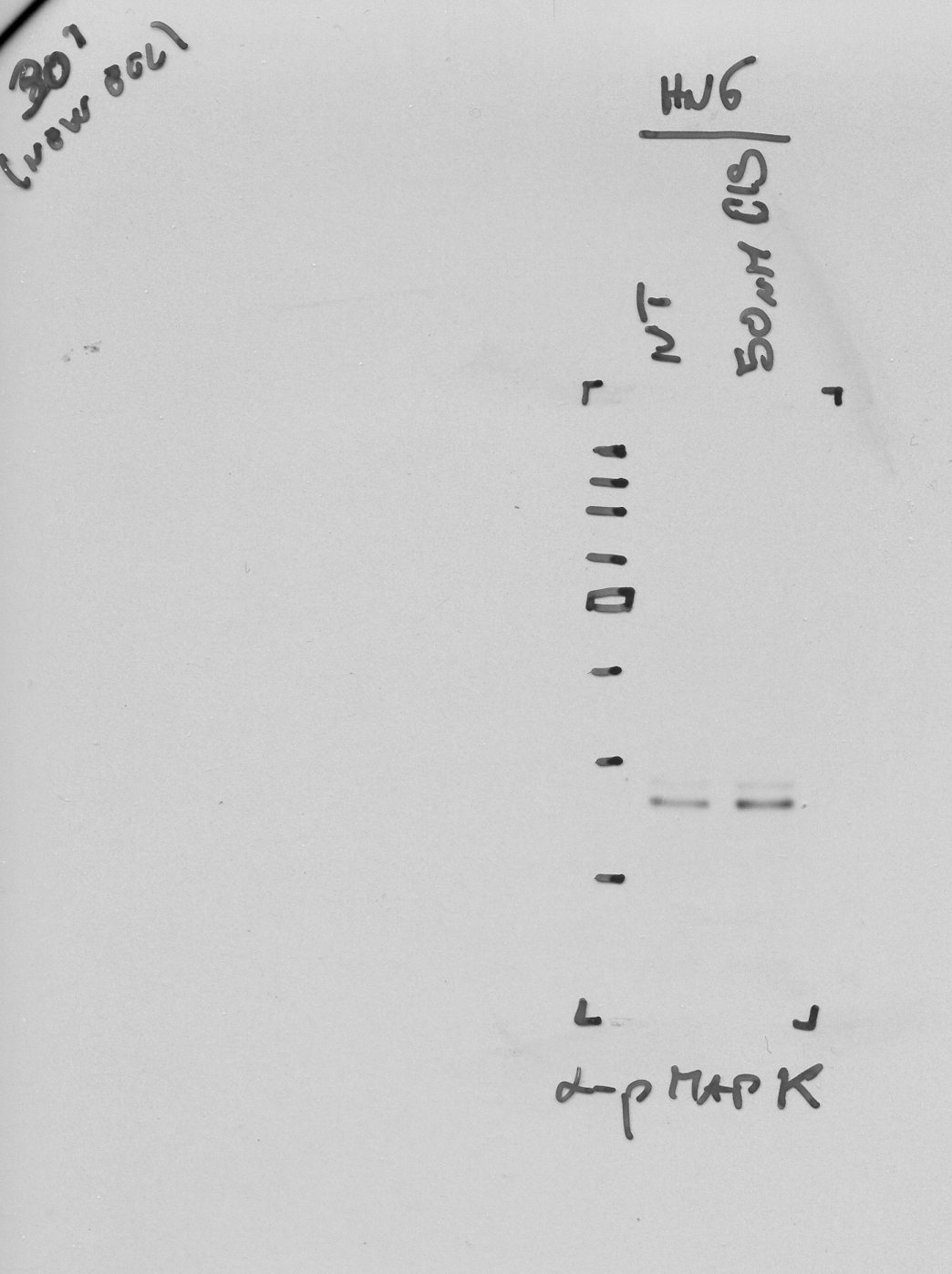

Supplement: Supplementary file 1 [file cancers-13-03822-s001.zip › Supplementary FIgure S1/Livelli189.jpg]

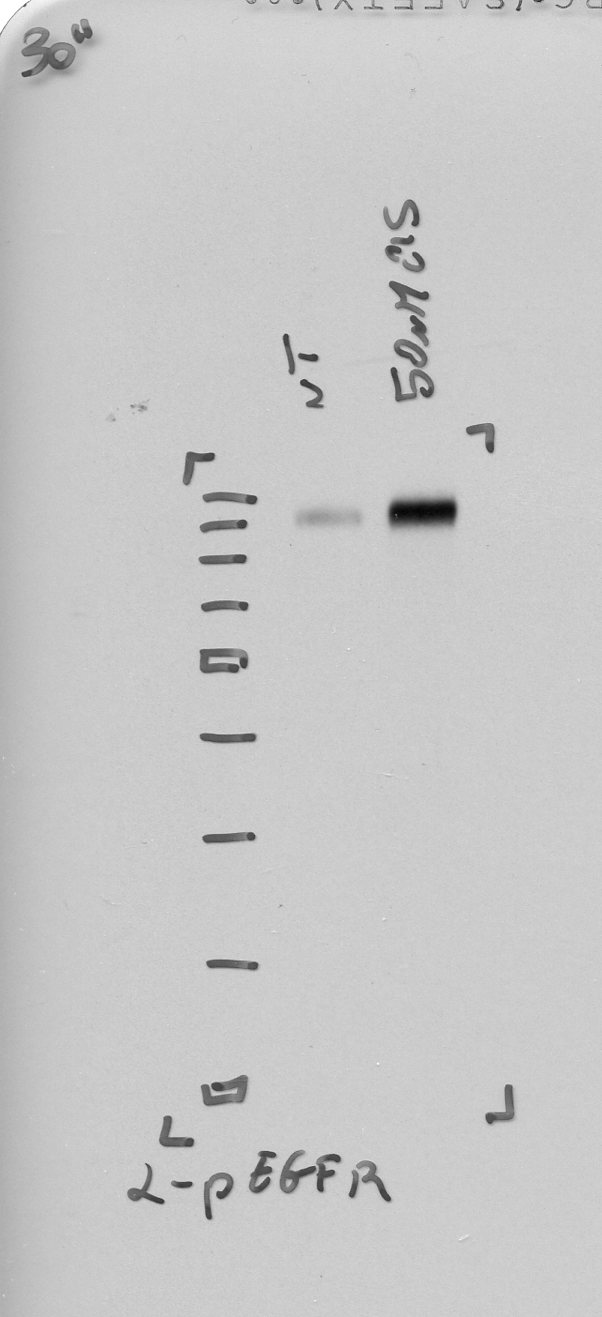

Supplement: Supplementary file 1 [file cancers-13-03822-s001.zip › Supplementary FIgure S1/Livelli193.jpg]

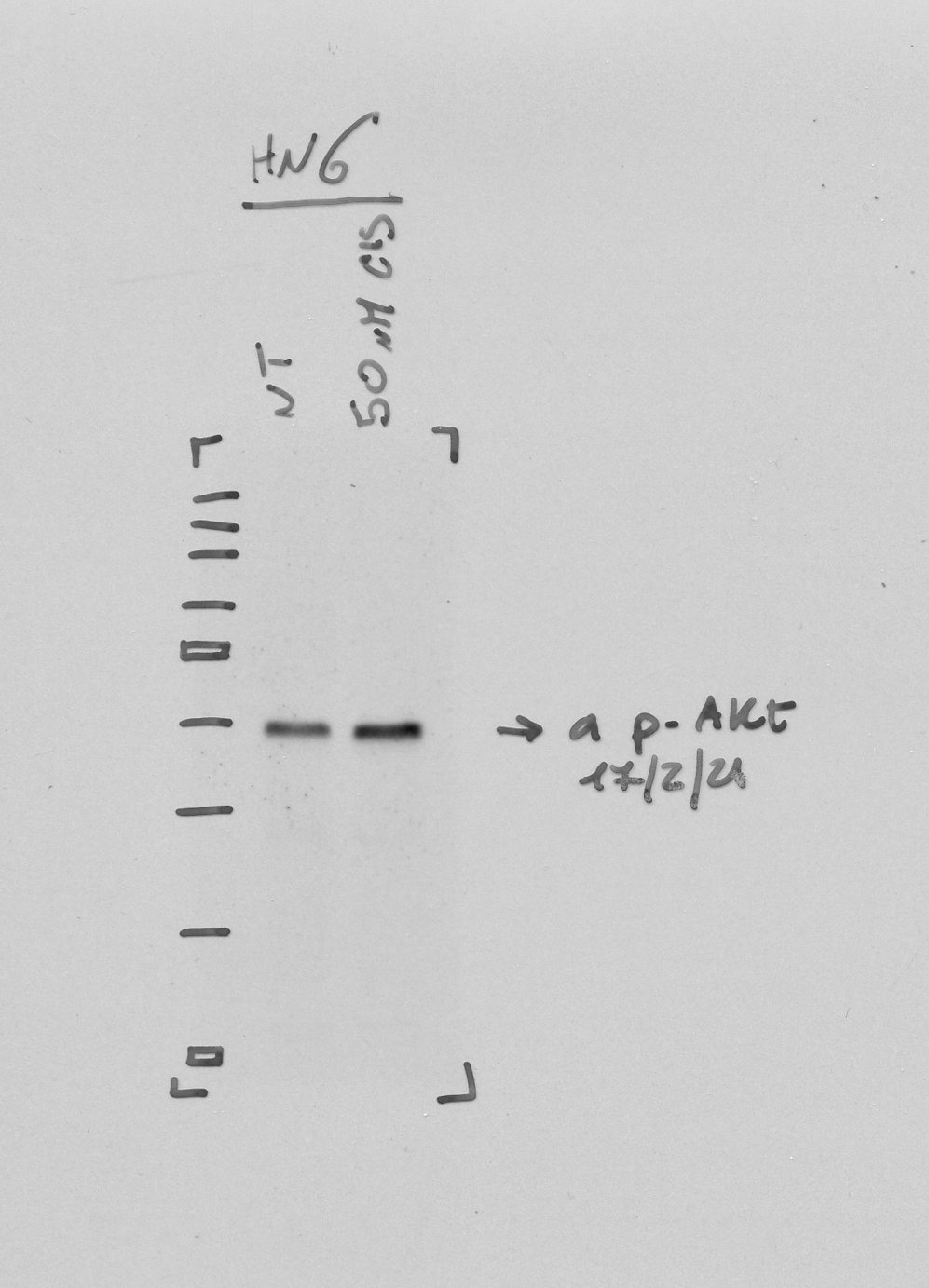

Supplement: Supplementary file 1 [file cancers-13-03822-s001.zip › Supplementary FIgure S1/Livelli196.jpg]

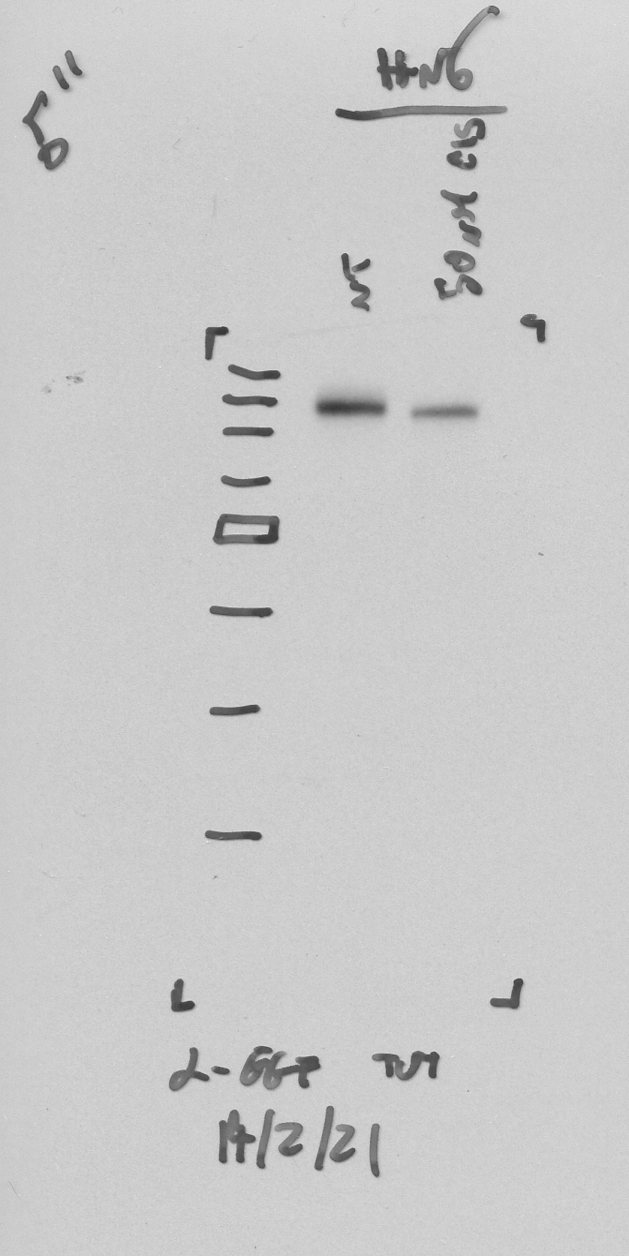

Supplement: Supplementary file 1 [file cancers-13-03822-s001.zip › Supplementary FIgure S1/Livelli198.jpg]

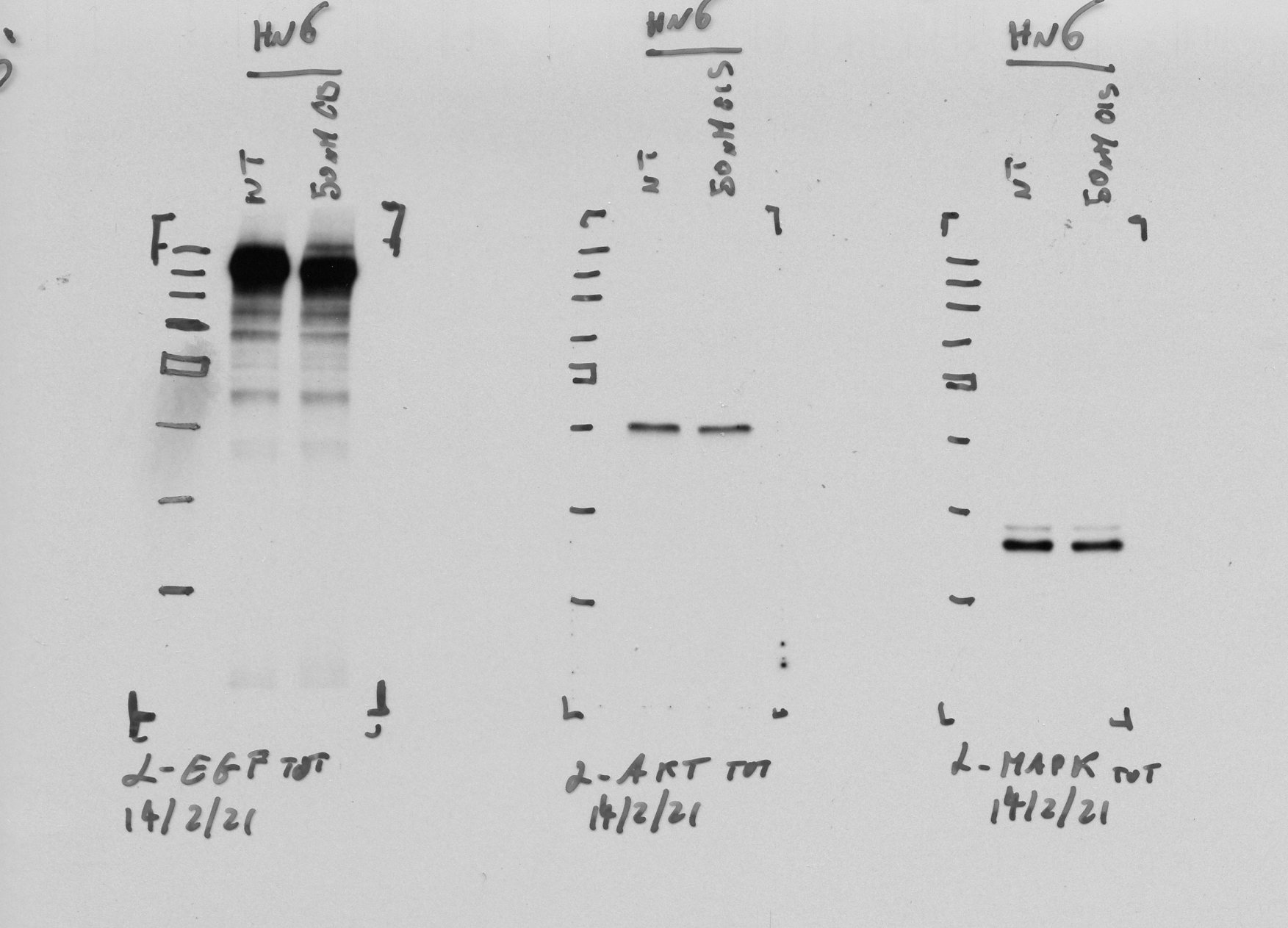

Supplement: Supplementary file 1 [file cancers-13-03822-s001.zip › Supplementary FIgure S1/Livelli201.jpg]
